# On the Rate-Distortion Function of Sampled Cyclostationary Gaussian Processes

**DOI:** 10.3390/e22030345

**Published:** 2020-03-17

**Authors:** Emeka Abakasanga, Nir Shlezinger, Ron Dabora

**Affiliations:** 1 Department of Electrical and Computer Engineering, Ben-Gurion University, Be’er-Sheva 8410501, Israel; abakasan@post.bgu.ac.il; 2 Faculty of Mathematics and Computer Science, Weizmann Institute of Science, Rehovot 7610001, Israel; nirshlezinger1@gmail.com

**Keywords:** wide-sense cyclostationary, wide-sense almost cyclostationary, rate-distortion function, information spectrum

## Abstract

Man-made communications signals are typically modelled as continuous-time (CT) wide-sense cyclostationary (WSCS) processes. As modern processing is digital, it is applied to discrete-time (DT) processes obtained by sampling the CT processes. When sampling is applied to a CT WSCS process, the statistics of the resulting DT process depends on the relationship between the sampling interval and the period of the statistics of the CT process: When these two parameters have a common integer factor, then the DT process is WSCS. This situation is referred to as synchronous sampling. When this is not the case, which is referred to as asynchronous sampling, the resulting DT process is wide-sense almost cyclostationary (WSACS). The sampled CT processes are commonly encoded using a source code to facilitate storage or transmission over wireless networks, e.g., using compress-and-forward relaying. In this work, we study the fundamental tradeoff between rate and distortion for source codes applied to sampled CT WSCS processes, characterized via the rate-distortion function (RDF). We note that while RDF characterization for the case of synchronous sampling directly follows from classic information-theoretic tools utilizing ergodicity and the law of large numbers, when sampling is asynchronous, the resulting process is not information stable. In such cases, the commonly used information-theoretic tools are inapplicable to RDF analysis, which poses a major challenge. Using the information-spectrum framework, we show that the RDF for asynchronous sampling in the low distortion regime can be expressed as the limit superior of a sequence of RDFs in which each element corresponds to the RDF of a synchronously sampled WSCS process (yet their limit is not guaranteed to exist). The resulting characterization allows us to introduce novel insights on the relationship between sampling synchronization and the RDF. For example, we demonstrate that, differently from stationary processes, small differences in the sampling rate and the sampling time offset can notably affect the RDF of sampled CT WSCS processes.

## 1. Introduction

Man-made signals are typically generated using a repetitive procedure, which takes place at fixed intervals. The resulting signals are thus commonly modeled as continuous-time (CT) random processes exhibiting periodic statistical properties [1,2,3], which are referred to as wide-sense cyclostationary (WSCS) processes. In digital communications, where the transmitted waveforms commonly obey the WSCS model [3], the received CT signal is first sampled to obtain a discrete-time (DT) received signal. In the event that the sampling interval is commensurate with the period of the statistics of the CT WSCS signal, cyclostationarity is preserved in DT ([3] Section 3.9). In this work, we refer to this situation as synchronous sampling. However, it is practically common to encounter scenarios in which the sampling rate at the receiver and the symbol rate of the received CT WSCS process are incommensurate, which is referred to as asynchronous sampling. The resulting sampled process in such cases is a DT wide-sense almost cyclostationary (WSACS) stochastic process ([3] Section 3.9).

This research aims at investigating lossy source coding for asynchronously sampled CT WSCS processes. In the source coding problem, every sequence of information symbols from the source is mapped into a sequence of code symbols, referred to as codewords, taken from a predefined codebook. In lossy source coding, the source sequence is recovered up to a predefined distortion constraint, within an arbitrary small tolerance of error. The figure-of-merit for lossy source coding is the rate-distortion function (RDF) which characterizes the minimum number of bits per source symbol required to compress the source sequence such that it can be reconstructed at the decoder within the specified maximal distortion [4]. For an independent and identically distributed (IID) random source process, the RDF can be expressed as the minimum mutual information between the source variable and the reconstruction variable, such that with the corresponding conditional distribution of the reconstruction symbol given the source symbol, the distortion constraint is satisfied ([5] Chapter 10). The source coding problem has been further studied in multiple different scenarios, including the reconstruction of a single source at multiple destinations [6] and the reconstruction of multiple correlated stationary Gaussian sources at a single destination [7,8,9].

For sampled stationary source processes, ergodicity theory and the asymptotic equipartition property (AEP) ([5] Chapter 3) were utilized for characterizing the RDF in different scenarios ([10] Chapter 9), ([4] Section I), [11]. However, as in a broad range of applications, including digital communications networks, the CT signals are WSCS processes, the sampling operation results in DT source signals whose statistics depends on the relationship between the sampling rate and the period of the statistics of the source signal. When sampling is synchronous, the resulting DT source signal is WSCS ([3] Section 3.9). The RDF for lossy compression of DT WSCS Gaussian sources with memory was studied in [12]. The work [12] used the fact that any WSCS signal can be transformed into a set of stationary subprocess [2]; thereby facilitating the application of information-theoretic results obtained for multivariate stationary sources to the derivation of the RDF; Nonetheless, in many digital communications scenarios, the sampling rate and the symbol rate of the CT WSCS process are not related in any way, and are possibly incommensurate, resulting in a sampled process which is a DT WSACS stochastic process. Such situations can occur as a result of the a-priori determined values of the sampling interval and the symbol duration of the WSCS source signal, as well as due to sampling clock jitters resulting from hardware impairments. A comprehensive review of trends and applications for almost cyclostationary signals can be found in [13]. Despite of their apparent frequent occurrences, the RDF for lossy compression of WSACS sources has not been characterized, which is the motivation for the current research. A major challenge associated with characterizing fundamental limits for asynchronously sampled WSCS processes stems from the fact that the resulting processes are not information stable, in the sense that their conditional distributions are not ergodic ([14] Page X), [15,16]. As a result, the standard information-theoretic tools cannot be employed, making the characterization of the RDF a very challenging problem.

Our recent study in [17] on channel coding reveals that for the case of additive CT WSCS Gaussian noise, capacity varies significantly with sampling rates, whether the Nyquist criterion is satisfied or not. In particular, it was observed that the capacity can change dramatically with minor variations in the sampling rate, causing it to switch from synchronous sampling to asynchronous sampling. This is in direct contrast to the results obtained for wide-sense stationary noise for which the capacity remains unchanged for any sampling rate above the Nyquist rate [18]. A natural fundamental question that arises from this result is how the RDF of a sampled Gaussian source process varies with the sampling rate. As a motivating example, one may consider compress-and-forward (CF) relaying, in which the relay samples at a rate which can be incommensurate with the symbol rate of the incoming communications signal.

In this work, we employ the information-spectrum framework [14] for characterizing the RDF of asynchronously sampled memoryless Gaussian WSCS processes, as this framework is applicable to the information-theoretic analysis of non information-stable processes ([14] Page VII). We further note that while rate characterizations obtained using information spectrum tools and its associated quantities may be difficult to evaluate ([14] Remark 1.7.3), here we obtain a numerically computable characterization of the RDF. In particular, we focus on the mean squared error (MSE) distortion measure in the low distortion regime, namely, source codes for which the average square of the difference between the source and the reproduction process is not larger than the minimal source variance. The results of this research lead to accurate modelling of signal compression in current and future digital communications systems. The derived RDF, which characterizes the fundamental performance limits in encoding sampled CT WSCS Gaussian processes into a digital representation, allows to evaluate source coding schemes associated with different levels of complexity in terms of their gap from optimality, when applied to this important class of signals.

Furthermore, we utilize our characterization of the RDF to examine how the RDF for a sampled CT WSCS Gaussian source varies with different sampling rates and sampling time offsets. We demonstrate that, differently from stationary sources, when applying a lossy source code to a sampled WSCS process, the achievable rate-distortion tradeoff can be significantly affected by minor variations in the sampling time offset and the sampling rate. Our results thus allow identifying the sampling rate and sampling time offsets which minimize the RDF in systems involving sampled WSCS processes.

The rest of this work is organised as follows: Section 2 provides a scientific background on cyclostationary processes and on rate-distortion analysis of DT WSCS Gaussian sources. Section 3 presents the problem formulation and auxiliary results, and Section 4 details the main result of RDF characterization for sampled WSCS Gaussian process. Numerical examples and discussions are provided in Section 5, and Section 6 concludes the paper.

## 2. Preliminaries and Background

In the following we review the main tools and framework used in this work: In Section 2.1 we detail the notations, and in Section 2.2 we review the basics of cyclostationary processes and the statistical properties of a DT process resulting from sampling a CT WSCS process. In Section 2.3 we recall some preliminaries of rate-distortion theory as well as the RDF for DT WSCS Gaussian source processes. This background creates a premise for the statement of the main result provided in Section 4 of this paper.

### 2.1. Notations

In this paper, random vectors are denoted by boldface uppercase letters, e.g., X; boldface lowercase letters denote deterministic column vectors, e.g., x. Scalar RVs and deterministic values are denoted via standard uppercase and lowercase fonts respectively, e.g., *X* and *x*. Scalar random processes are denoted with X(t),t∈R for CT and with X[n],n∈Z for DT. Uppercase Sans-Serif fonts represent matrices, e.g., A, and the element at the $i^{th}$ row and the $l^{th}$ column of A is denoted with ${\left(A\right)}_{i,l}$. We use |·| to denote the absolute value, ⌊d⌋,d∈R, to denote the floor function and $d^{+},d\in R$, to denote the max{0,d}. δ[·] denotes the Kronecker delta function: δ[n]=1 for n=0 and δ[n]=0 otherwise, and E{·} denotes the stochastic expectation. The sets of positive integers, integers, rational numbers, real numbers, positive numbers, and complex numbers are denoted by N,Z, Q, R, $R^{++}$, and C, respectively. The cumulative distribution function (CDF) is denoted by $F_{X}\left(x\right)≜\mathrm{Pr}\left(X\leq x\right)$ and the probability density function (PDF) of a CT random variable (RV) is denoted by $p_{X}\left(x\right)$. We represent a real Gaussian distribution with mean μ and variance $\sigma^{2}$ by the notation $N(\mu ,\sigma^{2})$. All logarithms are taken to base-2, and $j=\sqrt{-1}$. Lastly, for any sequence y[i], i∈N, and positive integer k∈N, $y^{\left(k\right)}$ denotes the column vector ${\left(y[1],\ldots ,y[k]\right)}^{T}$.

### 2.2. Wide-Sense Cyclostationary Random Processes

Here, we review some preliminaries from the theory of cyclostationarity. We begin by recalling the definition of wide-sense cyclostationary processes for CT and for DT:

**Definition** **1** (CT wide-sense cyclostationary processes ([3] Section 3.2.1))**.** *A scalar stochastic process* ${\left\{S\left(t\right)\right\}}_{t\in R}$ *is called WSCS if both its first-order and its second-order moments are periodic with respect to* t∈R *with some period* $T_{p}\in R$*.*

**Definition** **2** (DT wide-sense cyclostationary processes ([2] Section 17.2))**.** *A scalar stochastic process* ${\left\{S\left[n\right]\right\}}_{n\in Z}$ *is called WSCS if both its first-order and its second-order moments are periodic with respect to* n∈Z *with some period* $N_{p}\in Z$*.*

WSCS signal are thus random processes whose first and second-order moments are periodic functions with the same period. To define WSACS signals, we first recall the definition of almost-periodic functions:

**Definition** **3** (Almost-periodic functions ([19] Definition 2.1))**.** *A DT function* x[n]*,* n∈Z*, is called an almost-periodic function if for every* ϵ>0 *there exists a number* l(ϵ)∈N *with the property that for any* n∈Z *and any* α∈Z*,* $\exists \Delta \in \left[\alpha ,\alpha +l(\epsilon )\right]$*, such that*

|x[Δ]−x[n]|<ϵ.

**Definition** **4** (DT wide-sense almost-cyclostationary processes ([2] Section 17.2))**.** *A scalar stochastic process* ${\left\{S[n])\right\}}_{n\in Z}$ *is called WSACS if its first and its second order moments are almost-periodic functions with respect to* n∈Z*.*

**Remark** **1.** *Note that when the mean and the autocorrelation function are each periodic with periods which are incommensurate, the resulting processes is WSACS. We note that in many practical cases the mean is zero, see e.g., ([2] Section 17.2), hence the classification of the process will be determined by the periodicity of the autocorrelation function.*

The DT WSCS model is commonly used in the communications literature, as it facilitates the the analysis of many problems of interest, such as fundamental rate limits analysis [20,21,22], channel identification [23], synchronization [24], and noise mitigation [25]. However, in many scenarios, the considered signals are WSACS rather than WSCS. To see how the WSACS model is obtained in the context of sampled signals, we briefly recall the discussion in [17] on sampled WSCS processes (please refer to ([17] Section II.B) for more details): Consider a CT WSCS random process S(t), which is sampled uniformly with a sampling interval of $T_{s}$ and sampling time offset ϕ, resulting in a DT random process $S\left[i\right]=S\left(i\cdot T_{s}+\phi \right)$. It is well known that contrary to stationary processes, which have a time-invariant statistical characteristics, the values of $T_{s}$ and ϕ have a significant effect on the statistics of sampled WSCS processes ([17] Section II.B). To demonstrate this point, consider a CT WSCS process with variance $\sigma_{s}^{2}\left(t\right)=\frac{1}{2}\cdot \sin\left(2\pi t/T_{\mathrm{sym}}\right)+2$ for some $T_{\mathrm{sym}}>0$. The sampled process for ϕ=0 (no sampling time offset) and $T_{s}=\frac{T_{\mathrm{sym}}}{3}$ has a variance function whose period is $N_{p}=3$: $\sigma_{s}^{2}\left(iT_{s}\right)=\left\{2,2.433,1.567,2,2.433,1.567,\ldots \right\}$, for i=0,1,2,3,4,5,…; while the DT process obtained with the same sampling interval and with a sampling time offset of $\phi =\frac{T_{s}}{2\pi }$ has a periodic variance with $N_{p}=3$ with values $\sigma_{s}^{2}\left(iT_{s}+\phi \right)=\left\{2.155,2.335,1.510,2.155,2.335,1.510,\ldots \right\}$, for i=0,1,2,3,4,5,…, which are different from the values of the DT variance for ϕ=0. It follows that both variances are periodic in discrete-time with the same period $N_{p}=3$, although with different values within the period, which is a result of the sampling time offset, yet, both DT processes correspond to two instances of synchronous sampling. Lastly, consider the sampled variance obtained by sampling without a time offset (i.e., ϕ=0) at a sampling interval of $T_{s}=\left(1+\frac{1}{2\pi }\right)\frac{T_{\mathrm{sym}}}{3}$. For this case, $T_{s}$ is not an integer divisor of $T_{\mathrm{sym}}$ or of any of its integer multiples (i.e., $\frac{T_{\mathrm{sym}}}{T_{s}}=2+\frac{2\pi -2}{2\pi +1}\equiv 2+\epsilon$; where ϵ∉Q and ϵ∈[0,1)) resulting in the variance values $\sigma_{s}^{2}\left(iT_{s}\right)=\left\{2,2.335,1.5027,2.405,1.896,1.75,\ldots \right\}$, for i=0,1,2,3,4,5…. For this scenario, the DT variance is not periodic but is almost-periodic, corresponding to asynchronous sampling and the resulting DT process is not WSCS but WSACS ([3] Section 3.2). The example above demonstrates that the statistical properties of sampled WSCS processes depend on the sampling rate and the sampling time offset, implying that the RDF of such processes should also depend on these quantities, as we demonstrate in the sequel.

### 2.3. The Rate-Distortion Function for DT WSCS Processes

**Figure 1:**
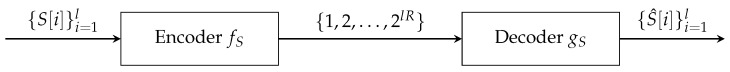
Source coding block diagram.

In this subsection we review the source coding problem and the existing results on the RDF of WSCS processes. We begin by recalling the definition of a source coding scheme, see, e.g., ([26] Chapter 30), ([5] Chapter 10):

**Definition** **5** (Source coding scheme)**.** *A source coding scheme with blocklength l consists of (see Figure 1):*

*1.*  *An encoder*$f_{S}$*which maps a block of l source samples*${\left\{S\left[i\right]\right\}}_{i=1}^{l}$*into an index from a set of*$M=2^{lR}$*indexes,*$f_{S}:{\left\{S\left[i\right]\right\}}_{i=1}^{l}\mapsto \left\{1,2,\ldots ,M\right\}$*.*
*2.*  *A decoder*$g_{S}$*which maps the received index into a reconstructed sequence of length l,*${\left\{\hat{S}\left[i\right]\right\}}_{i=1}^{l}$*,*$g_{S}:\left\{1,2,\ldots ,M\right\}\mapsto {\left\{\hat{S}\left[i\right]\right\}}_{i=1}^{l}$

*The encoder-decoder pair is referred to as an* (R,l) *source code, where R is the rate of the code in bits per source symbol, defined as:*

(1) $$R=\frac{1}{l}\log_{2}M$$

The RDF characterizes the minimal average number of bits per source symbol, denoted R(D), that can be used to encode a source process such that it can be reconstructed from its encoded representation with a recovery distortion not larger than D>0 ([5] Section 10.2). In the current work, we use the MSE distortion measure, which measures the distortion due to decoding a source symbol *S* into $\hat{S}$ via $d\left(S,\hat{S}\right)={\left(S-\hat{S}\right)}^{2}$. The distortion for a sequence of source samples $S^{\left(l\right)}$ decoded into a reproduction sequence ${\hat{S}}^{\left(l\right)}$ is given by $d\left(S^{\left(l\right)},{\hat{S}}^{\left(l\right)}\right)=\frac{1}{l}\sum_{i=1}^{l}{\left(S\left[i\right]-\hat{S}\left[i\right]\right)}^{2}$ and the average distortion in decoding a random source sequence $S^{\left(l\right)}$ into a random reproduction sequence ${\hat{S}}^{\left(l\right)}$ is defined as:

(2) $$\bar{d}\left(S^{\left(l\right)},{\hat{S}}^{\left(l\right)}\right)≜E\left\{d\left(S^{\left(l\right)},{\hat{S}}^{\left(l\right)}\right)\right\}=\frac{1}{l}\sum_{i=1}^{l}E\left\{{\left(S\left[i\right]-\hat{S}\left[i\right]\right)}^{2}\right\},$$

where the expectation in Equation (2) is taken with respect to the joint probability distributions on the source S[i] and its reproduction $\hat{S}\left[i\right]$. Using Definition 5 we can now define the achievable rate-distortion pair for a source S[i], as stated in the following definition ([10] Pg. 471):

**Definition** **6** (Achievable rate-distortion pair)**.** *A rate-distortion pair* (R,D) *is achievable for a process* ${\left\{S\left[i\right]\right\}}_{i\in N}$ *if for any* η>0 *and for all sufficiently large l it is possible to construct an* $\left(R_{s},l\right)$ *source code such that*

(3) $$R_{s}\leq R+\eta .$$

*and*

(4) $$\bar{d}\left(S^{\left(l\right)},{\hat{S}}^{\left(l\right)}\right)\leq D+\eta .$$

**Definition** **7.** *The rate-distortion function* R(D) *is defined as the infimum of all achievable rates R for a given maximum allowed distortion D.*

Definition 6 defines a rate-distortion pair to be achievable if the rate and the distortion constraints are satisfied using source codes with any sufficiently large blocklength. In the following lemma, which will be used to characterize the RDF of DT WSCS signals, we state that it is sufficient to consider only source codes whose blocklength is an integer multiple of some fixed positive integer:

**Lemma** **1.** *Consider the process* ${\left\{S\left[i\right]\right\}}_{i\in N}$ *with a finite and bounded variance. For a given maximum allowed distortion D, the optimal reproduction process* ${\left\{\hat{S}\left[i\right]\right\}}_{i\in N}$ *is also the optimal reproduction process when restricted to using source codes whose blocklengths are integer multiples of some fixed positive integer r.*

**Proof.**  The proof of the lemma is detailed in Appendix A. □

This lemma facilitates switching between multivariate and scalar representations of the source and the reproduction processes.

The RDF obviously depends on the distribution of the source ${\left\{S\left[i\right]\right\}}_{i\in N}$. Thus, statistically different sources have different RDFs. However, when a source is scaled by some positive constant, the RDF of the scaled process with the MSE distortion can be inferred from that of the original source process, as stated in the following theorem:

**Theorem** **1.** *Let* ${\left\{S\left[i\right]\right\}}_{i\in N}$ *be a source process for which the rate-distortion pair* (R,D) *is achievable under the MSE distortion. Then, for every* $\alpha \in R^{++}$ *, it holds that the rate-distortion pair* $\left(R,\alpha^{2}\cdot D\right)$ *is achievable for the scaled source* ${\left\{\alpha \cdot S\left[i\right]\right\}}_{i\in N}$ *.*

**Proof.**  The proof of the theorem is detailed in Appendix B. □

Lastly, in the proof of our main result, we make use of the RDF for DT WSCS sources derived in ([12] Theorem 1), repeated below for ease of reference. Prior to the statement of the theorem, we recall that for blocklenghts which are integer multiples of $N_{p}$, a WSCS process S[i] with period $N_{p}>0$ can be represented as an equivalent $N_{p}$-dimensional process $S^{\left(N_{p}\right)}\left[i\right]$ via the decimated component decomposition (DCD) ([2] Section 17.2). The power spectral density (PSD) of the process $S^{\left(N_{p}\right)}$ is defined as ([12] Section II):

(5) $${\left(\rho_{S}\left(e^{j2\pi f}\right)\right)}_{u,v}=\sum_{\Delta \in Z}{\left(R_{S}\left[\Delta \right]\right)}_{u,v}e^{-j2\pi f\Delta }\;-\frac{1}{2}\leq f\leq \frac{1}{2},\;u,v\in \left\{1,2,\ldots N_{p}\right\}$$

where $R_{S}\left[\Delta \right]≜E\left\{S^{\left(N_{p}\right)}\left[i\right]\cdot S^{\left(N_{p}\right)}\left[i+\Delta \right]\right\}$ ([2] Section 17.2). We now proceed to the statement of ([12] Theorem 1):

**Theorem** **2.** *([12] Theorem 1) Consider a zero-mean real DT WSCS Gaussian source* S[i],i∈N *with memory, and let* $N_{p}\in N$ *denote the period of its statistics. The RDF is expressed as:*

(6a) $$R\left(D\right)=\frac{1}{2N_{p}}\sum_{m=1}^{N_{p}}\int_{f=-0.5}^{0.5}{\left(\log\left(\frac{\lambda_{m}\left(e^{j2\pi f}\right)}{\theta }\right)\right)}^{+}df,$$

*where* $\lambda_{m}\left(e^{j2\pi f}\right)$ *,* $m=1,2,\ldots ,N_{p}$ *denote the eigenvalues of the PSD matrix of the process* $S^{\left(N_{p}\right)}\left[i\right]$ *, which is obtained from* S[i] *by applying the* $N_{p}$ *-dimensional DCD, and θ is selected such that*

(6b) $$D=\frac{1}{N_{p}}\sum_{m=1}^{N_{p}}\int_{f=-0.5}^{0.5}\min\left\{\lambda_{m}\left(e^{j2\pi f}\right),\theta \right\}df.$$

We note that $S^{\left(N_{p}\right)}\left[i\right]$ corresponds to a vector of stationary processes whose elements are not identically distributed; hence the variance function is different for each scalar stationary process. Using ([12] Theorem 1), we can directly obtain the RDF for the special case of a DT memoryless WSCS Gaussian process. This is stated in the following corollary:

**Corollary** **1.** *Let* ${\left\{S\left[i\right]\right\}}_{i\in N}$ *be a zero-mean DT memoryless real WSCS Gaussian source with period* $N_{p}\in N$ *, and set* $\sigma_{m}^{2}=E\left\{S^{2}\left[m\right]\right\}$ *for* $m=1,2,\ldots ,N_{P}$ *. The RDF for compression of* S[i] *is expressed as:*

(7a) $$\begin{array}{c} R\left(D\right)=\left\{\begin{array}{cc} \frac{1}{2N_{p}}\sum_{m=1}^{N_{p}}\log\left(\frac{\sigma_{m}^{2}}{D_{m}}\right) & D\leq \frac{1}{N_{p}}\sum_{m=1}^{N_{p}}\sigma_{m}^{2}\\ 0 & D>\frac{1}{N_{p}}\sum_{m=1}^{N_{p}}\sigma_{m}^{2}, \end{array}\right. \end{array}$$

*where* $D_{m}≜\min\left\{\sigma_{m}^{2},\theta \right\}$ *, and θ is defined such that*

(7b) $$D=\frac{1}{N_{p}}\sum_{m=1}^{N_{p}}D_{m}.$$

**Proof.**  Applying Equations (6a) and (6b) to our specific case of a memoryless WSCS source, we obtain Equations (7a) and (7b) as follows: First, note that the corresponding DCD components for a zero-mean memoryless WSCS process are also zero-mean and memoryless; hence the PSD matrix for the multivariate process $S^{\left(N_{p}\right)}\left[i\right]$ is a diagonal matrix, whose eigenvalues are the constant diagonal elements such that the *m*th diagonal element is equal to the variance $\sigma_{m}^{2}$: $\lambda_{m}\left(e^{j2\pi f}\right)=\sigma_{m}^{2}$. Now, writing Equation (6a) for this case we obtain:

(8) $$\begin{array}{cc} R(D) & =\frac{1}{2N_{p}}\sum_{m=1}^{N_{p}}\int_{f=-0.5}^{0.5}{\left(\log\left(\frac{\lambda_{m}\left(e^{j2\pi f}\right)}{\theta }\right)\right)}^{+}df\\ \; & =\frac{1}{2N_{p}}\sum_{m=1}^{N_{p}}{\left(\log\left(\frac{\sigma_{m}^{2}}{\theta }\right)\right)}^{+}. \end{array}$$

Since ${\left(\log\left(\frac{\sigma_{m}^{2}}{\theta }\right)\right)}^{+}=\max\left\{0,\log\left(\frac{\sigma_{m}^{2}}{\theta }\right)\right\}\equiv \log\left(\frac{\sigma_{m}^{2}}{D_{m}}\right)$ it follows that (8) coincides with (7a). Next, expressing Equation (6b) for the memoryless source process, we obtain:

(9) $$D=\frac{1}{N_{p}}\sum_{m=1}^{N_{p}}\int_{f=-0.5}^{0.5}\min\left\{\lambda_{m}\left(e^{j2\pi f}\right),\theta \right\}df=\frac{1}{N_{p}}\sum_{m=1}^{N_{p}}\min\left\{\sigma_{m}^{2},\theta \right\},$$

proving Equation (7b). □

Now, from Lemma 1, we conclude that the RDF for compression of source sequences whose blocklength is an integer multiple of $N_{p}$ is the same as the RDF for compressing source sequences whose blocklength is arbitrary. We recall that from ([5] Chapter 10.3.3) it follows that for the zero-mean memoryless Gaussian DCD vector source process $S^{\left(N_{p}\right)}\left[i\right]$ the optimal reproduction process which achieves the RDF is an $N_{p}\times 1$ memoryless process whose covariance matrix is diagonal with non-identically distributed elements. From [2], we can apply the inverse DCD to obtain a WSCS process. Hence, from Lemma 1 we can conclude that the optimal reproduction process for the DT WSCS Gaussian source is a DT WSCS Gaussian process.

## 3. Problem Formulation and Auxiliary Results

Our objective is to characterize the RDF for compression of asynchronously sampled CT WSCS Gaussian sources when the sampling interval is larger than the memory of the source. In particular, we focus on the minimal rate required to achieve a high fidelity reproduction, representing the RDF curve for distortion values not larger than the variance of the source. Such characterization of the RDF for asynchronous sampling is essential for comprehending the relationship between the minimal required number of bits and the sampling rate at a given distortion. Our analysis constitutes an important step towards constructing joint source-channel coding schemes for scenarios in which the symbol rate of the transmitter is not necessarily synchronized with the sampling rate of the source to be transmitted. Such scenarios arise, for example, when recording a communications signal for storage or processing, or in compress-and-forward relaying (([26] Chapter 16.7), [27]) in which the relay compresses the sampled received signal, which is then forwarded to the assisted receiver. As the relay operates with its own sampling clock, which need not necessarily be synchronized with the symbol rate of the assisted transmitter, sampling at the relay may result in a DT WSACS source signal. In the following we first characterize the sampled source model in Section 3.1. Then, as a preliminary step towards our characterization the RDF for asynchronously sampled CT WSCS Gaussian processes stated in Section 4, we recall in Section 3.2 the definitions of some information-spectrum quantities used in this study. Finally, in Section 3.3, we recall an auxiliary result relating the information-spectrum quantities of a collection of sequences of RVs to the information-spectrum quantities of its limit sequence of RVs. This result will be applied in the derivation of the RDF with asynchronous sampling.

### 3.1. Source Model

Consider a real CT, zero-mean WSCS Gaussian random process $S_{c}\left(t\right)$ with period $T_{\mathrm{ps}}$. Let the variance function of $S_{c}\left(t\right)$ be defined as $\sigma_{S_{c}}^{2}\left(t\right)≜E\left\{S_{c}^{2}\left(t\right)\right\}$, and assume it is both upper bounded and lower bounded away from zero, and that it is continuous in t∈R. Let $\tau_{m}>0$ denote the maximal correlation length of $S_{c}\left(t\right)$, i.e., $r_{S_{c}}\left(t,\tau \right)≜E\left\{S_{c}\left(t\right)S_{c}\left(t-\tau \right)\right\}=0,\forall \left|\tau \right|>\tau_{m}$. By the cyclostationarity of $S_{c}\left(t\right)$, we have that $\sigma_{S_{c}}^{2}\left(t\right)=\sigma_{S_{c}}^{2}\left(t+T_{\mathrm{ps}}\right),\forall t\in R$. Let $S_{c}\left(t\right)$ be sampled uniformly with the sampling interval $T_{s}>0$ such that $T_{\mathrm{ps}}=\left(p+\epsilon \right)\cdot T_{s}$ for p∈N and ϵ∈[0,1) yielding $S_{\epsilon }\left[i\right]≜S_{c}\left(i\cdot T_{s}\right)$, where i∈Z. The variance of $S_{\epsilon }\left[i\right]$ is given by $\sigma_{S_{\epsilon }}^{2}\left[i\right]≜r_{S_{\epsilon }}\left[i,0\right]=\sigma_{S_{c}}^{2}\left(\frac{i\cdot T_{\mathrm{ps}}}{p+\epsilon }\right)$.

In this work, as in [17], we assume that the duration of temporal correlation of the CT signal is shorter than the sampling interval $T_{s}$, namely, $\tau_{m}<T_{s}$. Consequently, the DT Gaussian process $S_{\epsilon }\left[i\right]$ is a memoryless zero-mean Gaussian process and its autocorrelation function is given by:

(10) $$\begin{array}{cc} r_{S_{\epsilon }}\left[i,\Delta \right] & =E\left\{S_{\epsilon }\left[i\right]S_{\epsilon }\left[i+\Delta \right]\right\}\\ & =E\left\{S_{c}\left(\frac{i\cdot T_{\mathrm{ps}}}{p+\epsilon }\right)\cdot S_{c}\left(\frac{\left(i+\Delta \right)\cdot T_{\mathrm{ps}}}{p+\epsilon }\right)\right\}=\sigma_{S_{c}}^{2}\left(\frac{i\cdot T_{\mathrm{ps}}}{p+\epsilon }\right)\cdot \delta \left[\Delta \right]=\sigma_{S_{\epsilon }}^{2}\left[i\right]\cdot \delta \left[\Delta \right]. \end{array}$$

While we do not explicitly account for sampling time offsets in our definition of the sampled process $S_{\epsilon }\left[i\right]$, it can be incorporated by replacing $\sigma_{S_{c}}^{2}\left(t\right)$ with a time-shifted version, i.e., $\sigma_{S_{c}}^{2}\left(t-\phi \right)$, see also ([17] Section II.C).

It can be noted from (10) that if ϵ is a rational number, i.e., ∃u,v∈N, *u* and *v* are relatively prime, such that $\epsilon =\frac{u}{v}$, then ${\left\{S_{\epsilon }\left[i\right]\right\}}_{i\in Z}$ is a DT memoryless WSCS process with the period $p_{u,v}=p\cdot v+u\in N$ ([17] Section II.C). For this class of processes, the RDF can be obtained from ([12] Theorem 1) as stated in Corollary 1. On the other hand, if ϵ is an irrational number, then sampling becomes asynchronous and leads to a WSACS process whose RDF has not been characterized to date.

### 3.2. Definitions of Relevant Information-Spectrum Quantities

Conventional information theoretic tools for characterizing RDFs are based on an underlying ergodicity of the source. Consequently, these techniques cannot be applied to characterize the RDF of asynchronously sampled WSCS processes. To tackle this challenge, we use the information-spectrum framework, as this framework [14] can be utilized to obtain general formulas for rate limits for any arbitrary class of processes. The resulting expressions are not restricted to specific statistical models of the considered processes, and in particular, do not require information stability or stationarity. In the following, we recall the definitions of several information-spectrum quantities used in this study, see also ([14] Definitions 1.3.1 and 1.3.2):

**Definition** **8.** *The limit-inferior in probability of a sequence of real RVs* ${\left\{Z_{k}\right\}}_{k\in N}$ *is defined as*

(11) $$p-\underset{k\to \infty }{\lim\inf}Z_{k}≜\sup\left\{\alpha \in R|\lim_{k\to \infty }\mathrm{Pr}\left(Z_{k}<\alpha \right)=0\right\}≜\alpha_{0}.$$

Hence, $\alpha_{0}$ is the largest real number satisfying that $\forall \tilde{\alpha }<\alpha_{0}$ and ∀μ>0 there exists $k_{0}\left(\mu ,\tilde{\alpha }\right)\in N$ such that $\mathrm{Pr}(Z_{k}<\tilde{\alpha })<\mu$, $\forall k>k_{0}\left(\mu ,\tilde{\alpha }\right)$.

**Definition** **9.** *The limit-superior in probability of a sequence of real RVs* ${\left\{Z_{k}\right\}}_{k\in N}$ *is defined as*

(12) $$p-\underset{k\to \infty }{\lim\sup}Z_{k}≜\inf\left\{\beta \in R|\lim_{k\to \infty }\mathrm{Pr}\left(Z_{k}>\beta \right)=0\right\}≜\beta_{0}.$$

Hence, $\beta_{0}$ is the smallest real number satisfying that $\forall \tilde{\beta }>\beta_{0}$ and ∀μ>0, there exists $k_{0}\left(\mu ,\tilde{\beta }\right)\in N$, such that $\mathrm{Pr}(Z_{k}>\tilde{\beta })<\mu$, $\forall k>k_{0}\left(\mu ,\tilde{\beta }\right)$.

The notion of uniform integrability of a sequence of RVs is a basic property in probability ([28] Chapter 12), which is not directly related to information spectrum methods. However, since it plays an important role in the information spectrum characterization of RDFs, we include its statement in the following definition:

**Definition** **10** (Uniform integrability ([28] Definition 12.1), ([14] Equation (5.3.2)))**.** *The sequence of real-valued random variables* ${\left\{Z_{k}\right\}}_{k=1}^{\infty }$*, is said to satisfy uniform integrability if*

(13) $$\lim_{u\to \infty }\underset{k\geq 1}{\sup}\int_{z:|z|\geq u}p_{Z_{k}}\left(z\right)\left|z\right|dz=0$$

The aforementioned quantities facilitate characterizing the RDF of arbitrary sources. Consider a general source process ${\left\{S\left[i\right]\right\}}_{i=1}^{\infty }$ (stationary or non-stationary) taking values from the source alphabet S[i]∈S and a reproduction process ${\left\{\hat{S}\left[i\right]\right\}}_{i=1}^{\infty }$ with values from the reproduction alphabet $\hat{S}\left[i\right]\in \hat{S}$. It follows from ([14] Section 5.5) that for a distortion measure which satisfies the uniform integrability criterion, i.e., that there exists a deterministic sequence ${\left\{r\left[i\right]\right\}}_{i=1}^{\infty }$ such that the sequence of RVs ${\left\{d\left(S^{\left(k\right)},r^{\left(k\right)}\right)\right\}}_{k=1}^{\infty }$ satisfies Definition 10 ([14] Page 336), then the RDF is expressed as ([14] Equation (5.4.2)):

(14) $$R\left(D\right)=\underset{F_{S,\hat{S}}:{\bar{d}}_{S}\left(S^{\left(k\right)},{\hat{S}}^{\left(k\right)}\right)\leq D}{\inf}\bar{I}\left(S^{\left(k\right)};{\hat{S}}^{\left(k\right)}\right),$$

where ${\bar{d}}_{S}\left(S^{\left(k\right)},{\hat{S}}^{\left(k\right)}\right)=\underset{k\to \infty }{\lim\sup}E\left\{d\left(S^{\left(k\right)},{\hat{S}}^{\left(k\right)}\right)\right\}$, $F_{S,\hat{S}}$ denotes the joint CDF of ${\left\{S\left[i\right]\right\}}_{i=1}^{\infty }$ and ${\left\{\hat{S}\left[i\right]\right\}}_{i=1}^{\infty }$, and $\bar{I}\left(S^{\left(k\right)}:{\hat{S}}^{\left(k\right)}\right)$ represents the limit superior in probability of the mutual information rate of $S^{\left(k\right)}$ and ${\hat{S}}^{\left(k\right)}$, given by:

(15) $$\bar{I}\left(S^{\left(k\right)};{\hat{S}}^{\left(k\right)}\right)≜p-\underset{k\to \infty }{\lim\sup}\frac{1}{k}\log\frac{p_{S^{\left(k\right)}|{\hat{S}}^{\left(k\right)}}\left(S^{\left(k\right)}|{\hat{S}}^{\left(k\right)}\right)}{p_{S^{\left(k\right)}}\left(S^{\left(k\right)}\right)}$$

In order to use the RDF characterization in (14), the distortion measure must satisfy the uniform integrability criterion. For the considered class of sources detailed in Section 3.1, the MSE distortion satisfies this criterion, as stated in the following lemma:

**Lemma** **2.** *For any real memoryless zero-mean Gaussian source* ${\left\{S\left[i\right]\right\}}_{i=1}^{\infty }$ *with bounded variance, i.e.,* $\exists \sigma_{\max}^{2}<\infty$ *such that* $E\left\{S^{2}\left[i\right]\right\}\leq \sigma_{\max}^{2}$ *for all* i∈N *, the MSE distortion satisfies the uniform integrability criterion.*

**Proof.**  Set the deterministic sequence ${\left\{r\left[i\right]\right\}}_{i=1}^{\infty }$ to be the all-zero sequence. Under this setting and the MSE distortion, it holds that $d\left(S^{\left(k\right)},r^{\left(k\right)}\right)=\frac{1}{k}\sum_{i=1}^{k}S^{2}\left[i\right]$. To prove the lemma, we show that the sequence of RVs ${\left\{d\left(S^{\left(k\right)},r^{\left(k\right)}\right)\right\}}_{k=1}^{\infty }$ has a bounded $\ell_{2}$ norm, which implies that it is uniformly integrable by ([28] Corollary 12.8). The $\ell_{2}$ norm of $d\left(S^{\left(k\right)},r^{\left(k\right)}\right)$ satisfies

(16) $$\begin{array}{cc} E\left\{d{\left(S^{\left(k\right)},r^{\left(k\right)}\right)}^{2}\right\} & =\frac{1}{k^{2}}E\left\{\sum_{i=1}^{k}S^{2}\left[i\right]\sum_{j=1}^{k}S^{2}\left[j\right]\right\}\\ \; & =\frac{1}{k^{2}}\sum_{i=1}^{k}\sum_{j=1}^{k}E\left\{S^{2}\left[i\right]S^{2}\left[j\right]\right\}\overset{\left(a\right)}{\leq }\frac{1}{k^{2}}\sum_{i=1}^{k}\sum_{j=1}^{k}3\sigma_{\max}^{4}=3\sigma_{\max}^{4}, \end{array}$$

where (a) follows since $E\left\{S^{2}\left[i\right]S^{2}\left[j\right]\right\}=E\left\{S^{2}\left[i\right]\right\}E\left\{S^{2}\left[j\right]\right\}=\sigma_{\max}^{4}$ for i≠j while $E\left\{S^{4}\left[i\right]\right\}=3\sigma_{\max}^{4}$ ([29] Chapter 5.4). Equation (16) proves that $d\left(S^{\left(k\right)},r^{\left(k\right)}\right)$ is $\ell_{2}$-bounded by $3\sigma_{\max}^{4}<\infty$ for all k∈N, which in turn implies that the MSE distortion is uniformly integrable for the source ${\left\{S\left[i\right]\right\}}_{i=1}^{\infty }$. □

Since, as detailed in Section 3.1, we focus in the following on memoryless zero-mean Gaussian sources, Lemma 2 implies that the RDF of the source can be characterized using (14). However, (14) is in general difficult to evaluate, and thus does not lead to a meaningful understanding of how the RDF of sampled WSCS sources behaves, motivating our analysis in Section 4.

### 3.3. Information Spectrum Limits

The following theorem originally stated in ([17] Theorem 1) presents a fundamental result which is directly useful for the derivation of the RDF:

**Theorem** **3.** *([17] Theorem 1) Let* ${\left\{{\tilde{Z}}_{k,n}\right\}}_{n,k\in N}$ *be a set of sequences of real scalar RVs satisfying two assumptions:*

*AS1*  *For every fixed* n∈N *, every convergent subsequence of* ${\left\{{\tilde{Z}}_{k,n}\right\}}_{k\in N}$ *converges in distribution, as* k→∞ *, to a finite deterministic scalar. Each subsequence may converge to a different scalar.*
*AS2*  *For every fixed* k∈N *, the sequence* ${\left\{{\tilde{Z}}_{k,n}\right\}}_{n\in N}$ *converges uniformly in distribution, as* n→∞ *, to a scalar real-valued RV* $Z_{k}$ *. Specifically, letting* ${\tilde{F}}_{k,n}\left(\alpha \right)$ *and* $F_{k}\left(\alpha \right)$ *,* α∈R *, denote the CDFs of* ${\tilde{Z}}_{k,n}$ *and of* $Z_{k}$ *, respectively, then by AS2 it follows that* ∀η>0 *, there exists* $n_{0}\left(\eta \right)$ *such that for every* $n>n_{0}\left(\eta \right)$
  $$\left|{\tilde{F}}_{k,n}\left(\alpha \right)-F_{k}\left(\alpha \right)\right|<\eta ,$$
  *for each* α∈R *,* k∈N *.*

*Then, for* ${\left\{{\tilde{Z}}_{k,n}\right\}}_{n,k\in N}$ *it holds that*

(17a) $$\begin{array}{ccc} p-\underset{k\to \infty }{\lim\inf}Z_{k} & = & \lim_{n\to \infty }\left(p-\underset{k\to \infty }{\lim\inf}{\tilde{Z}}_{k,n}\right), \end{array}$$

(17b) $$\begin{array}{ccc} p-\underset{k\to \infty }{\lim\sup}Z_{k} & = & \lim_{n\to \infty }\left(p-\underset{k\to \infty }{\lim\sup}{\tilde{Z}}_{k,n}\right). \end{array}$$

**Proof.**  In Appendix C we explicitly prove Equation (17b). This complements the proof in ([17] Appendix A) which explicitly considers only (17a). □

## 4. Rate-Distortion Characterization for Sampled CT WSCS Gaussian Sources

### 4.1. Main Result

Using the information-spectrum based characterization of the RDF (14) combined with the characterization of the limit of a sequence of information spectrum quantities in Theorem 3, we now analyze the RDF of asynchronously sampled WSCS processes. Our analysis is based on constructing a sequence of synchronously sampled WSCS processes, whose RDF is given in Corollary 1. Then, we show that the RDF of the asynchronously sampled process can be obtained as the limit superior of the computable RDFs of the sequence of synchronously sampled processes. We begin by letting $\epsilon_{n}≜\frac{\left\lfloor n\cdot \epsilon \right\rfloor }{n}$ for n∈N and defining a Gaussian source process $S_{n}\left[i\right]=S_{c}\left(\frac{i\cdot T_{\mathrm{ps}}}{p+\epsilon_{n}}\right)$. From the discussion in Section 3.1 (see also ([17] Section II.C)), it follows that since $\epsilon_{n}$ is rational, $S_{n}\left[i\right]$ is a WSCS process and its period is given by $p_{n}=p\cdot n+\left\lfloor n\cdot \epsilon \right\rfloor$. Accordingly, the periodic correlation function of $S_{n}\left[i\right]$ can be obtained similarly to (10) as:

(18) $$r_{S_{n}}\left[i,\Delta \right]=E\left\{S_{n}\left[i\right]S_{n}\left[i+\Delta \right]\right\}=\sigma_{S_{c}}^{2}\left(\frac{i\cdot T_{\mathrm{ps}}}{p+\epsilon_{n}}\right)\cdot \delta \left[\Delta \right].$$

Due to cyclostationarity of $S_{n}\left[i\right]$, we have that $r_{S_{n}}\left[i,\Delta \right]=r_{S_{n}}\left[i+p_{n},\Delta \right]$, ∀i,Δ∈Z, and we let $\sigma_{S_{n}}^{2}\left[i\right]≜r_{S_{n}}\left[i,0\right]$ denote its periodic variance.

We next restate Corollary 1 in terms of $\epsilon_{n}$ as follows:

**Proposition** **1.** *Consider a DT, memoryless, zero-mean, WSCS Gaussian random process* $S_{n}\left[i\right]$ *with a variance* $\sigma_{S_{n}}^{2}\left[i\right]$ *, obtained from* $S_{c}\left(t\right)$ *by sampling with a sampling interval of* $T_{s}\left(n\right)=\frac{T_{\mathrm{ps}}}{p+\epsilon_{n}}$ *. Let* $S_{n}^{\left(p_{n}\right)}\left[i\right]$ *denote the memoryless stationary multivariate random process obtained by applying the DCD to* $S_{n}\left[i\right]$ *and let* $\sigma_{S_{n}}^{2}\left[m\right]$ *,* $m=1,2,\ldots ,p_{n}$ *, denote the variance of the* $m^{th}$ *component of* $S_{n}^{\left(p_{n}\right)}\left[i\right]$ *. The rate-distortion function is given by:*

(19a) $$\begin{array}{c} R_{n}\left(D\right)=\left\{\begin{array}{cc} \frac{1}{2p_{n}}\sum_{m=1}^{p_{n}}\left(\log\left(\frac{\sigma_{S_{n}}^{2}\left[m\right]}{D_{n}\left[m\right]}\right)\right) & D\leq \frac{1}{p_{n}}\sum_{m=1}^{p_{n}}\sigma_{S_{n}}^{2}\left[m\right]\\ 0 & D>\frac{1}{p_{n}}\sum_{m=1}^{p_{n}}\sigma_{S_{n}}^{2}\left[m\right] \end{array}\right., \end{array}$$

*where for* $D\leq \frac{1}{p_{n}}\sum_{m=1}^{p_{n}}\sigma_{S_{n}}^{2}\left[m\right]\;$ *we let* $D_{n}\left[m\right]≜\min\left\{\sigma_{S_{n}}^{2}\left[m\right],\theta_{n}\right\}$ *, and* $\theta_{n}$ *is selected such that*

(19b) $$D=\frac{1}{p_{n}}\sum_{m=1}^{p_{n}}D_{n}\left[m\right].$$

We recall that the RDF of $S_{n}\left[i\right]$ is characterized in Proposition 1 via the RDF of the multivariate stationary process $S_{n}^{\left(p_{n}\right)}\left[i\right]$ obtained via a $p_{n}$-dimensional DCD applied to $S_{n}\left[i\right]$. Next, we recall that the relationship between the source process $S_{n}^{\left(p_{n}\right)}\left[i\right]$ and the optimal reconstruction process, denoted by ${\hat{S}}_{n}^{\left(p_{n}\right)}\left[i\right]$, is characterized in ([5] Chapter 10.3.3) via a linear, multivariate, time-invariant backward channel with a $p_{n}\times 1$ additive vector noise process $W_{n}^{\left(p_{n}\right)}\left[i\right]$, and is given by:

(20) $$S_{n}^{\left(p_{n}\right)}\left[i\right]={\hat{S}}_{n}^{\left(p_{n}\right)}\left[i\right]+W_{n}^{\left(p_{n}\right)}\left[i\right],\;i\in N.$$

It also follows from ([5] Section 10.3.3) that for the IID Gaussian multivariate process whose entries are independent and distributed via ${\left(S_{n}^{\left(p_{n}\right)}\left[i\right]\right)}_{m}∼N\left(0,\sigma_{S_{n}}^{2}\left[m\right]\right)$, $m\in \{1,2,\ldots ,p_{n}\}$, the optimal reconstruction vector process ${\hat{S}}_{n}^{\left(p_{n}\right)}\left[i\right]$ and the corresponding noise vector process $W_{n}^{\left(p_{n}\right)}\left[i\right]$ each follow a multivariate Gaussian distribution:

$${\hat{S}}_{n}^{\left(p_{n}\right)}\left[i\right]∼N\left(0,\left[\begin{array}{ccc} \sigma_{{\hat{S}}_{n}}^{2}\left[1\right] & \cdots & 0\\ \vdots & \ddots & \vdots \\ 0 & \cdots & \sigma_{{\hat{S}}_{n}}^{2}\left[p_{n}\right] \end{array}\right]\right)\;\mathrm{and}\;W_{n}^{\left(p_{n}\right)}\left[i\right]∼N\left(0,\left[\begin{array}{ccc} D_{n}\left[1\right] & \cdots & 0\\ \vdots & \ddots & \vdots \\ 0 & \cdots & D_{n}\left[p_{n}\right] \end{array}\right]\right),$$

where $D_{n}\left[m\right]≜\min\left\{\sigma_{S_{n}}^{2}\left[m\right],\theta_{n}\right\}$; $\theta_{n}$ denotes the reverse waterfilling threshold defined in Prop. 1 for the index *n*, and is selected such that $D=\frac{1}{p_{n}}\sum_{m=1}^{p_{n}}D_{n}\left[m\right]$. The optimal reconstruction process, ${\hat{S}}_{n}^{\left(p_{n}\right)}\left[i\right]$ and the noise process $W_{n}^{\left(p_{n}\right)}\left[i\right]$ are mutually independent, and for each $m\in \{1,2,\ldots ,p_{n}\}$ it holds that $E\left\{{\left(S_{n}^{\left(p_{n}\right)}\left[i\right]-{\hat{S}}_{n}^{\left(p_{n}\right)}\left[i\right]\right)}_{m}^{2}\right\}=D_{n}\left[m\right]$, see ([5] Chapters 10.3.2 and 10.3.3). The multivariate relationship between stationary processes in (20) can be transformed into an equivalent linear relationship between cyclostationary Gaussian memoryless processes via the inverse DCD transformation ([2] Sec 17.2) applied to each of the processes, resulting in:

(21) $$S_{n}\left[i\right]={\hat{S}}_{n}\left[i\right]+W_{n}\left[i\right],\;i\in N.$$

We are now ready to state our main result, which is the RDF of asynchronously sampled DT sources $S_{\epsilon }\left[i\right],\epsilon \notin Q$, in the low MSE regime, i.e., when the distortion *D* is not larger than the source variance. The RDF is stated in the following theorem, which applies to both synchronous sampling as well as to asynchronous sampling:

**Theorem** **4.** *Consider a DT source* ${\left\{S_{\epsilon }\left[i\right]\right\}}_{i=1}^{\infty }$ *obtained by sampling a CT WSCS source, whose period of statistics is* $T_{\mathrm{ps}}$ *, at intervals* $T_{s}$ *. Then, for any distortion constraint D such that* $D<\min_{0\leq t\leq T_{\mathrm{ps}}}\sigma_{S_{c}}^{2}\left(t\right)$ *and any* ϵ∈[0,1) *, the RDF* $R_{\epsilon }\left(D\right)$ *for compressing* ${\left\{S_{\epsilon }\left[i\right]\right\}}_{i=1}^{\infty }$ *can be obtained as the limit:*

(22) $$R_{\epsilon }\left(D\right)=\underset{n\to \infty }{\lim\sup}R_{n}\left(D\right),$$

*where* $R_{n}\left(D\right)$ *is defined Prop. 1.*

**Proof.**  The detailed proof is provided in Appendix D. Here, we give a brief outline: The derivation of the RDF with asynchronous sampling follows three steps: First, we note that sampling at a rate of $T_{s}\left(n\right)=\frac{T_{\mathrm{ps}}}{p+\epsilon_{n}}$ results in a sequence of DT WSCS sources ${\left\{S_{n}\left[i\right]\right\}}_{i\in N,n\in N}$ whose sampling interval $T_{s}\left(n\right)$ asymptotically approaches, as n→∞, the sampling interval for irrational ϵ given by $T_{s}=\frac{T_{\mathrm{ps}}}{p+\epsilon }$. We define a sequence of rational numbers $\epsilon_{n}$s.t.$\epsilon_{n}\to \epsilon$ as n→∞; Building upon this insight, we prove that the RDF with $T_{s}$ can be stated as a double limit where the outer limit is with respect to the blocklength and the inner limit is with respect to $\epsilon_{n}$. Lastly, we use Theorem 3 to show that the order of the limits can be exchanged, obtaining a limit of expressions which are computable. □

**Remark** **2.** *Theorem 4 focuses on the low distortion regime, defined as the values of D satisfying* $D<\min_{0\leq t\leq T_{\mathrm{ps}}}\sigma_{S_{c}}^{2}\left(t\right)$ *. This implies that* $\theta_{n}$ *has to be smaller than* $\min_{0\leq t\leq T_{\mathrm{ps}}}\sigma_{S_{c}}^{2}\left(t\right)$ *; hence, from Prop. 1 it follows that for the corresponding stationary noise vector* $W_{n}^{\left(p_{n}\right)}\left[i\right]$ *in *(20)*,* $D_{n}\left[m\right]=\min\left\{\sigma_{S_{n}}^{2}\left[m\right],\theta_{n}\right\}=\theta_{n}$ *and* $D=\frac{1}{p_{n}}\sum_{m=1}^{p_{n}}D_{n}\left[m\right]=\theta_{n}=D_{n}\left[m\right]$ *. We note that since every element of the vector* ${\left(W_{n}^{\left(p_{n}\right)}\left[i\right]\right)}_{m}$ *has the same variance* $D_{n}\left[m\right]=D$ *for all* n∈N *and* $m=1,2,\ldots ,p_{n}$ *then by applying the inverse DCD to* $W_{n}^{\left(p_{n}\right)}\left[i\right]$ *, the resulting scalar DT process* $W_{n}\left[i\right]$ *is wide sense stationary; and in fact IID with* $E\left\{{\left(W_{n}\left[i\right]\right)}^{2}\right\}=D$ *.*

### 4.2. Discussion and Relationship with Capacity Derivation in Reference 17

Theorem 4 provides a meaningful and computable characterization for the RDF of sampled WSCS signals. We note that the proof of the main theorem uses some of the steps used in our recent study on the capacity of memoryless channels with sampled CT WSCS Gaussian noise [17]. It should be emphasized, however, that there are several fundamental differences between the two studies, which require the introduction of new treatments and derivations original to the current work. First, it is important to note that in the study on capacity, a physical channel model exists, and therefore the conditional PDF of the output signal given the input signal can be characterized explicitly for both synchronous sampling and asynchronous sampling for every input distribution. For the current study of the RDF we note that the relationship (21), commonly referred to as the backward channel [30], ([5] Chapter 10.3.2), characterizes the relationship between the source process and the optimal reproduction process, and hence is valid only for synchronous sampling and for the optimal reproduction process. Consequently, in the RDF analysis the limiting relationship (21) as n→∞ is not even known to exist and, in fact, we can show it exists under a rather strict condition on the distortion (namely, the condition $D<\min_{0\leq t\leq T_{\mathrm{ps}}}\sigma_{S_{c}}^{2}\left(t\right)$ stated in Theorem 4). In particular, to prove the statement in Theorem 4, we had to show that from the backward channel (21), we can define an asymptotic relationship, as n→∞, which corresponds to the asynchronously sampled source process, denoted by $S_{\epsilon }\left[i\right]$, and relates $S_{\epsilon }\left[i\right]$ with its optimal reconstruction process ${\hat{S}}_{\epsilon }\left[i\right]$. This is done by showing that the PDFs for the reproduction process ${\hat{S}}_{n}\left[i\right]$ and noise process $W_{n}\left[i\right]$ from (21), each converge uniformly as n→∞ to a respective limiting PDF, which has to be defined as well. This enabled us to relate the RDFs for the synchronous sampling and for the asynchronous sampling cases using Theorem 3, eventually leading to (22). Accordingly, in our detailed proof of Theorem 4 given in Appendix D, Lemmas A6 and A8 as well as a significant part of Lemma A4 are largely new, addressing the special aspects of the proof arising from the fundamental differences between current setup and the setup in [17], while the derivations of Lemmas A3 and A7 follow similarly to ([17] Lemma B.1) and ([17] Lemma B.5), respectively, and parts of Lemma A4 coincide with ([17] Lemma B.2).

## 5. Numerical Examples

In this section we demonstrate the insights arising from our RDF characterization via numerical examples. Recalling that Theorem 4 states the RDF for asynchronously sampled CT WSCS Gaussian process, $R_{\epsilon }\left(D\right)$, as the limit supremum of a sequence of RDFs corresponding to DT memoryless WSCS Gaussian source processes ${\left\{R_{n}\left(D\right)\right\}}_{n\in N}$, we first consider the convergence of ${\left\{R_{n}\left(D\right)\right\}}_{n\in N}$ in Section 5.1. Next, in Section 5.2 we study the variation of the RDF of the sampled CT process due to changes in the sampling rate and in the sampling time offset.

Similarly to ([17] Section IV), define a periodic continuous pulse function, denoted by $\Pi_{t_{\mathrm{dc}},t_{\mathrm{rf}}}\left(t\right)$, with equal rise/fall time $t_{\mathrm{rf}}=0.01$, duty cycle $t_{\mathrm{dc}}\in \left[0,0.98\right]$, and period of 1, i.e., $\Pi_{t_{\mathrm{dc}},t_{\mathrm{rf}}}\left(t+1\right)=\Pi_{t_{\mathrm{dc}},t_{\mathrm{rf}}}\left(t\right)$ for all t∈R. Specifically, for t∈[0,1) the function $\Pi_{t_{\mathrm{dc}},t_{\mathrm{rf}}}\left(t\right)$ is given by

(23) $$\Pi_{t_{\mathrm{dc}},t_{\mathrm{rf}}}\left(t\right)=\left\{\begin{array}{cc} \frac{t}{t_{\mathrm{rf}}} & t\in [0,t_{\mathrm{rf}}]\\ 1 & t\in (t_{\mathrm{rf}},t_{\mathrm{dc}}+t_{\mathrm{rf}})\\ 1-\frac{t-t_{\mathrm{dc}}-t_{\mathrm{rf}}}{t_{\mathrm{rf}}} & t\in [t_{\mathrm{dc}}+t_{\mathrm{rf}},t_{\mathrm{dc}}+2\cdot t_{\mathrm{rf}}]\\ 0 & t\in (t_{\mathrm{dc}}+2\cdot t_{\mathrm{rf}},1). \end{array}\right.$$

In the following, we model the time varying variance of the WSCS source $\sigma_{S_{c}}^{2}\left(t\right)$ to be a linear periodic function of $\Pi_{t_{\mathrm{dc}},t_{\mathrm{rf}}}\left(t\right)$. To that aim, we define a time offset between the first sample and the rise start time of the periodic continuous pulse function; we denote the time offset by ϕ∈[0,1). This corresponds to the sampling time offset normalized to the period $T_{\mathrm{ps}}$. The variance of $S_{c}\left(t\right)$ is a periodic function with period $T_{\mathrm{ps}}$ which is defined as

(24) $$\sigma_{S_{c}}^{2}\left(t\right)=0.2+4.8\cdot \Pi_{t_{\mathrm{dc}},t_{\mathrm{rf}}}\left(\frac{t}{T_{\mathrm{ps}}}-\phi \right),\;t\in \left[0,T_{\mathrm{ps}}\right),$$

with a period of $T_{\mathrm{ps}}=5$ μsecs.

### 5.1. Convergence of $R_{n}\left(D\right)$ in n

From Theorem 4 it follows that if the distortion satisfies $D<\min_{0\leq t\leq T_{\mathrm{ps}}}\sigma_{S_{c}}^{2}\left(t\right)$, the RDF of the asynchronously sampled CT WSCS Gaussian process is given by the limit superior of the sequence ${\left\{R_{n}\left(D\right)\right\}}_{n\in N}$; where $R_{n}\left(D\right)$ is defined in Proposition 1. In this subsection, we study the sequence of RDFs ${\left\{R_{n}\left(D\right)\right\}}_{n\in N}$ as *n* increases. For this evaluation setup, we fixed the distortion constraint at D=0.18 and set $\epsilon =\frac{\pi }{7}$ and p=2. Let the variance of the CT WSCS Gaussian source process $\sigma_{S_{c}}^{2}\left(t\right)$ be modelled by Equation (24) for two sampling time offsets $\phi =\{0,\frac{1}{16}\}$. For each offset ϕ, four duty cycle values were considered: $t_{\mathrm{dc}}=\left[20,45,75,98\right]\%$. For each *n* we obtain the synchronous sampling mismatch $\epsilon_{n}≜\frac{\left\lfloor n\cdot \epsilon \right\rfloor }{n}$, which approaches ϵ as n→∞, where n∈N. Since $\epsilon_{n}$ is a rational number, corresponding to a sampling period of $T_{s}\left(n\right)=\frac{T_{\mathrm{ps}}}{p+\epsilon_{n}}$, then for each *n*, the resulting dt process is WSCS with the period $p_{n}=p\cdot n+\left\lfloor n\cdot \epsilon \right\rfloor$ and its RDF follows from Proposition 1.

**Figure 2:**
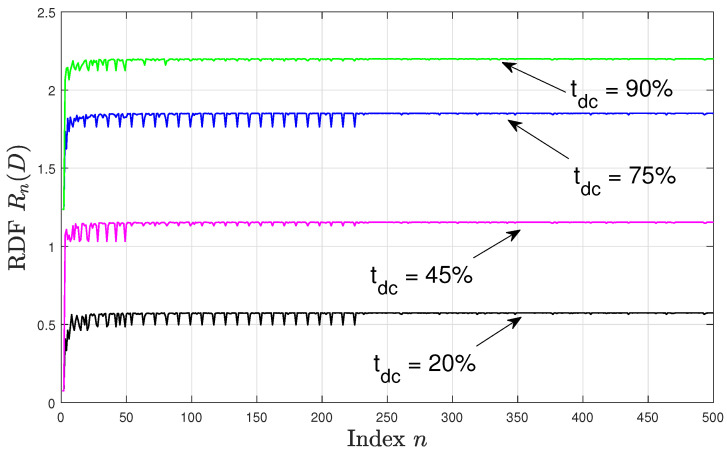
$R_{n}\left(D\right)$ versus *n*; offset ϕ=0.

**Figure 3:**
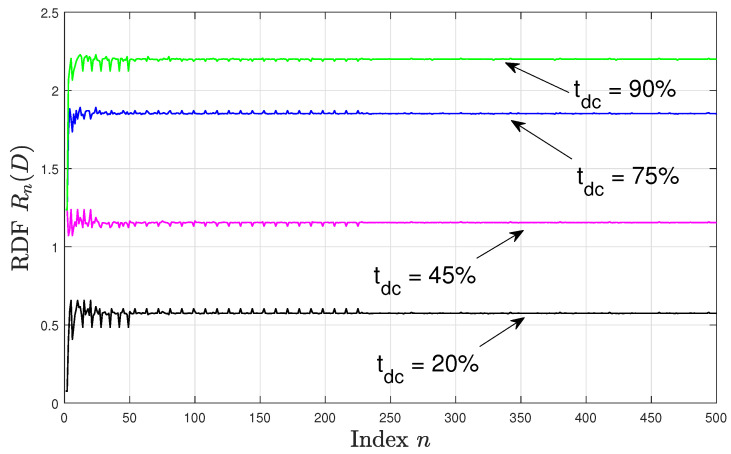
$R_{n}\left(D\right)$ versus *n*; offset $\phi =\frac{1}{16}$.

Figure 2 and Figure 3 depict $R_{n}\left(D\right)$ for n∈[1,500] with the specified duty cycles and sampling time offsets, where in Figure 2 there is no sampling time offset, i.e., ϕ=0, and in Figure 3 the sampling time offset is set to $\phi =\frac{1}{16}$. We observe that in both figures the RDF values are higher for higher $t_{\mathrm{dc}}$. This can be explained by noting that for higher $t_{\mathrm{dc}}$ values, the resulting time-averaged variance of the DT source process increases, hence, a higher number of bits per source sample is required to encode the source process maintaining the same distortion value. Moreover, in all configurations, $R_{n}\left(D\right)$ varies significantly for smaller values of *n*. Comparing Figure 2 and Figure 3, we see that the pattern of these variations depends on the sampling time offset ϕ. For example, when $t_{\mathrm{dc}}=45\%$ at n∈[4,15], then for ϕ=0 the rdf varies in the range [1.032,1.143] bits per source sample, while for $\phi =\frac{1}{16}$ the RDF varies in the range [1.071,1.237] bits per source sample. However, as *n* increases above 230, the variations in $R_{n}\left(D\right)$ become smaller and are less dependent on the sampling time offset, and the resulting values of $R_{n}\left(D\right)$ are approximately in the same range for each $t_{\mathrm{dc}}$ in both Figure 2 and Figure 3 for n≥230. This behaviour can be explained by noting that as *n* varies, the period $p_{n}$ also varies and hence the statistics of the DT variance differs over its respective period. This consequently affects the resulting RDF (especially for small periods). As *n* increases $\epsilon_{n}$ approaches the asynchronous sampling mismatch ϵ and the period $p_{n}$ takes a sufficiently large value such that the samples of the DT variance over the period are identically distributed irrespective of the value of ϕ; leading to a negligible variation in the RDF as seen in the above figures.

### 5.2. The Variation of the RDF with the Sampling Rate

**Figure 4:**
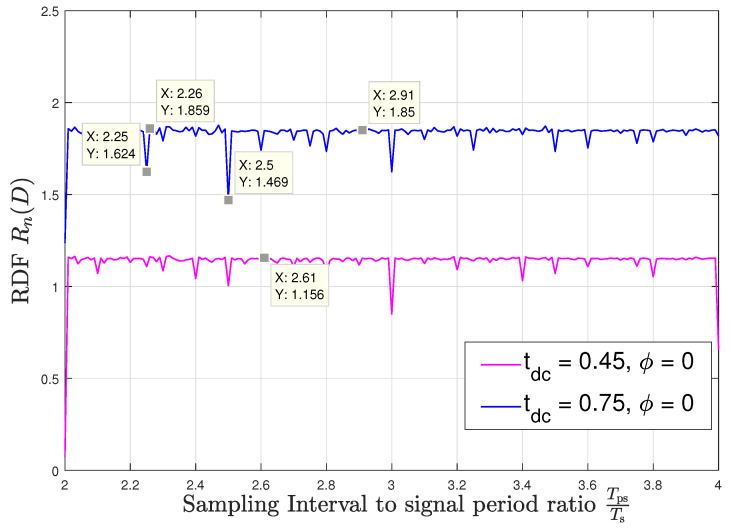
$R_{n}\left(D\right)$ versus $\frac{T_{\mathrm{ps}}}{T_{s}}$; offset ϕ=0.

**Figure 5:**
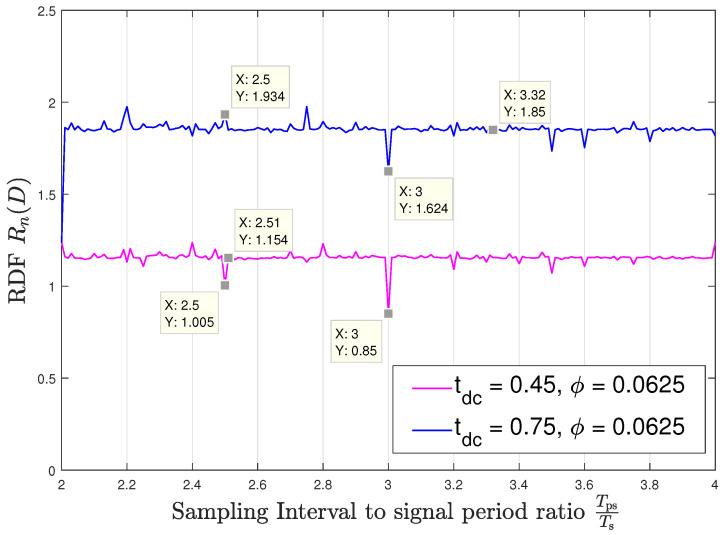
$R_{n}\left(D\right)$ versus $\frac{T_{\mathrm{ps}}}{T_{s}}$; offset $\phi =\frac{1}{16}$.

Next, we observe the dependence of the RDF for the sampled memoryless WSCS Gaussian process on the value of the sampling interval $T_{s}$. For this setup, we fix the distortion constraint to D=0.18 and set the duty cycle in the source process (24) to $t_{\mathrm{dc}}=\left[45,75\right]\%$. Figure 4 and Figure 5 demonstrate the numerically evaluated values for $R_{n}\left(D\right)$ at sampling intervals in the range $2<\frac{T_{\mathrm{ps}}}{T_{s}}<4$ with the sampling time offsets ϕ=0 and $\phi =\frac{1}{16}$, respectively. We note that while the discussion which follows focuses on this range, as it corresponds to relatively low sampling rates—which are typically preferable in practice, the statements and observations regarding the relationship between the denominator of $\frac{T_{\mathrm{ps}}}{T_{s}}$ and the value of $R_{n}\left(D\right)$, and regarding the continuity the RDF in the parameter $\frac{T_{\mathrm{ps}}}{T_{s}}$, are directly applicable to any range of values of $\frac{T_{\mathrm{ps}}}{T_{s}}$, e.g., when higher sampling rates are preferable. A very important insight which arises from the figures is that the sequence of RDFs $R_{n}\left(D\right)$ is not convergent; hence, for example, one cannot approach the RDF for $\frac{T_{\mathrm{ps}}}{T_{s}}=2.5$ by simply taking rational values of $\frac{T_{\mathrm{ps}}}{T_{s}}$ which approach 2.5. This verifies that the RDF for asynchronous sampling cannot be obtained by straightforward application of previous results, and indeed, the entire analysis carried out in the manuscript is necessary for the desired characterization.

We observe in Figure 4 and Figure 5 that when $\frac{T_{\mathrm{ps}}}{T_{s}}$ has a fractional part with a relatively small integer denominator, the variations in the RDF are significant and depend on the sampling time offset. These variations can either degrade the ability to accurately represent the source, which are the observed peaks in Figure 4 and Figure 5, or alternatively, allow to encode the signal to within the same distortion with smaller code rates, corresponding to the deeps in these figures. However, when $\frac{T_{\mathrm{ps}}}{T_{s}}$ approaches an irrational number, the period of the sampled variance function becomes very long, and consequently, the RDF is approximately constant and independent of the sampling time offset. As an example, consider $\frac{T_{\mathrm{ps}}}{T_{s}}=2.5$ and $t_{\mathrm{dc}}=75\%$: For sampling time offset ϕ=0 the rdf takes a value of 1.469 bits per source sample, as shown in Figure 4 while for the offset of $\phi =\frac{1}{16}$ the RDF peaks to 1.934 bits per source sample as can be seen in Figure 5. On the other hand, when approaching asynchronous sampling, the RDF takes a nearly constant value of 1.85 bits per source sample for all the considered values of $\frac{T_{\mathrm{ps}}}{T_{s}}$ and this value is invariant to the offset ϕ. This follows since when the denominator of the fractional part of $\frac{T_{\mathrm{ps}}}{T_{s}}$ increases, then the DT period of the resulting sampled variance, $p_{n}$, increases and practically captures the entire set of values of the CT variance regardless of the sampling time offset. In a similar manner as with the study on capacity in [17], we conjecture that since asynchronous sampling captures the entire set of values of the CT variance, the respective RDF represents the RDF of the analog source, which does not depend on the specific sampling rate and offset. Figure 4 and Figure 5 demonstrate how slight variations in the sampling rate can result in significant changes in the RDF. For instance, at ϕ=0 we observe in Figure 4 that when the sampling rate switches from $T_{s}=2.25\cdot T_{\mathrm{ps}}$ to $T_{s}=2.26\cdot T_{\mathrm{ps}}$, i.e., the sampling rate switches from being synchronous to being nearly asynchronous, then the RDF changes from 1.624 bits per source sample to 1.859 bits per source sample for $t_{\mathrm{dc}}=75\%$; also, we observe in Figure 5 for $t_{\mathrm{dc}}=45\%$, that when the sampling rate switches from $T_{s}=2.5\cdot T_{\mathrm{ps}}$ to $T_{s}=2.51\cdot T_{\mathrm{ps}}$, i.e., the sampling rate also switches from being synchronous to being nearly asynchronous, then the rdf changes from 1.005 bits per source sample to 1.154 bits per source sample.

**Figure 6:**
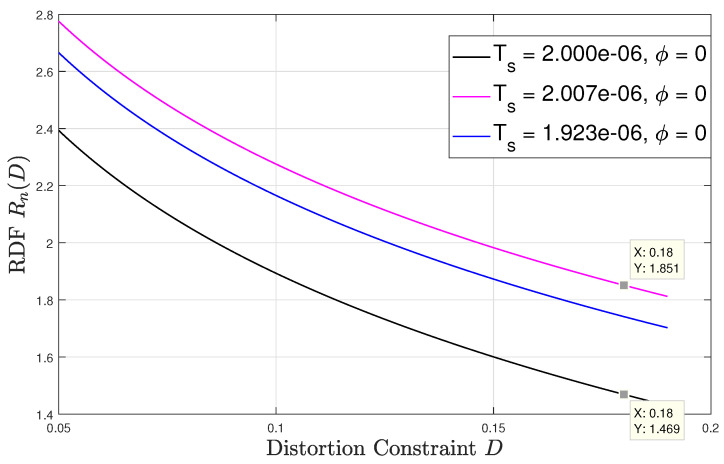
$R_{n}\left(D\right)$ versus *D*; offset ϕ=0.

**Figure 7:**
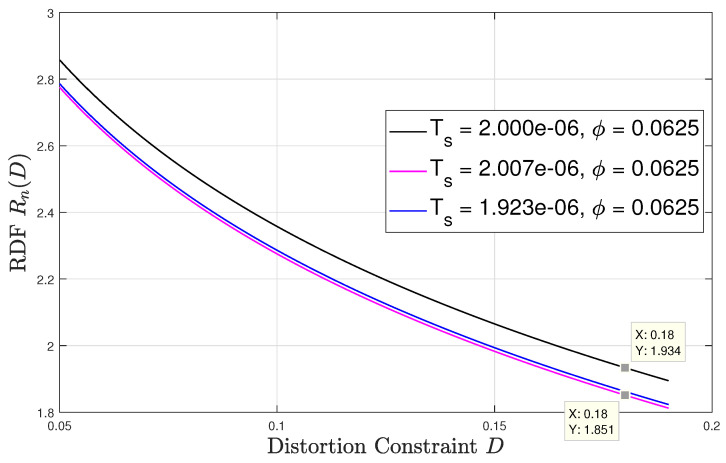
$R_{n}\left(D\right)$ versus *D*; offset $\phi =\frac{1}{16}$.

**Figure 8:**
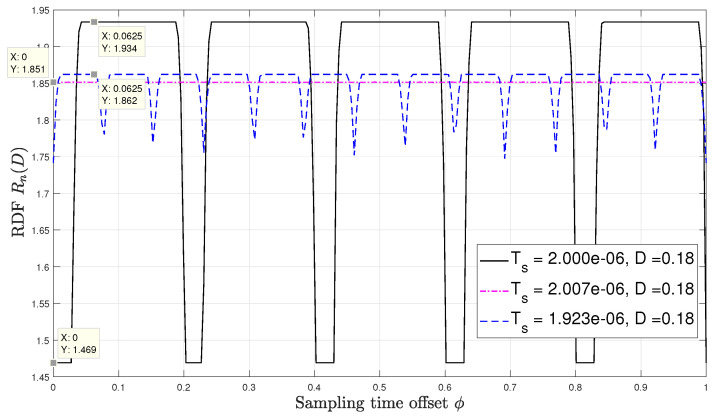
$R_{n}\left(D\right)$ versus ϕ at $t_{\mathrm{dc}}=75\%$.

Lastly, Figure 6 and Figure 7 numerically evaluate the RDF versus the distortion constraint D∈[0.05,0.19] for sampling time offsets of 0 and $\frac{1}{16}$ respectively. At each ϕ, the result is evaluated at three different values of synchronization mismatch ϵ. For this setup, we fix $t_{\mathrm{dc}}=75\%$, p=2 and $\epsilon \in \{0.5,\frac{5\pi }{32},0.6\}$. The only mismatch value that refers to the asynchronous sampling case is $\epsilon =\frac{5\pi }{32}$ and its corresponding sampling interval is approximately 2.007 μsecs, which is a negligible variation from the sampling intervals corresponding to ϵ∈{0.5,0.6}, which are 2.000 μsecs and 1.923 μsecs, respectively. Observing both figures, we see that the rdf may vary significantly for very slight variation in the sampling rate. For instance, as shown in Figure 6 for ϕ=0, at D=0.18, a slight change in the synchronization mismatch from $\epsilon =\frac{5\pi }{32}$ (i.e., $T_{s}\approx 2.007\mu$secs) to ϵ=0.5 (i.e., $T_{s}=2.000\;\mu$secs) results to approximately 20% decrease in the rdf. For $\phi =\frac{1}{16}$ the same change in the sampling synchronization mismatch at D=0.18 results in an increase in the RDF by roughly 4%. These results demonstrate the unique and counter-intuitive characteristics of the RDF of sampled WSCS signals which arise from our derivation. It is also interesting to examine how the RDF varies with the sampling time offset ϕ. To that aim we plot in Figure 8 the RDF vs. ϕ for the three sampling rates used in Figure 6 and Figure 7 at D=0.18. The points marked on the plot correspond to ϕ=0 and $\phi =\frac{1}{16}$ considered in Figure 6 and Figure 7, respectively. We observe that the RDF is indeed periodic with ϕ. These variations in the RDF occur as by changing ϕ the number of high variance samples within a period of the variance of the DT process changes due to the duty cycle of the CT variance. Then, at ϕ=0 the periodic variance of the DT process corresponding to $T_{s}=2.000\mu \mathrm{secs}$ has the smallest number of high variance values within a period, and when $\phi =\frac{1}{16}$ the periodic variance of the DT process corresponding to asynchronous sampling has the smallest number of high variance values within a period. For the asynchronous sampling rate the sampling time offset does not matter as in any case (nearly) all values of the CT variance are reflected in the variance of the DT process.

## 6. Conclusions

In this work the RDF of a sampled CT WSCS Gaussian source process was characterized for scenarios in which the resulting DT process is memoryless and the distortion is relatively small. This characterization shows the relationship between the sampling rate and the minimal number of bits per source sample required for compression at a given distortion. For cases in which the sampling rate is synchronized with the period of the statistics of the source process, the resulting DT process is WSCS and standard information theoretic framework can be used for deriving its RDF. For asynchronous sampling, information stability does not hold, and hence we resort to the information spectrum framework to obtain a characterization. To that aim we derived a relationship between some relevant information spectrum quantities for uniformly convergent sequences of RVs. This relationship was further applied to characterize the RDF of an asynchronously sampled CT WSCS Gaussian source process as the limit superior of a sequence of RDFs, each corresponding to the synchronous sampling of the CT WSCS Gaussian process. The results were derived in the low distortion regime, i.e., under the condition that the distortion constraint *D* is less than the minimum variance of the source, and for sampling intervals which are larger than the correlation length of the CT process. Our numerical examples give rise to non-intuitive insights which follow from the derivations. In particular, the numerical evaluation demonstrates that the RDF for a sampled CT WSCS Gaussian source can change dramatically with minor variations in the sampling rate and the sampling time offset. In particular, when the sampling rate switches from being synchronous to being asynchronous and vice versa, the RDF may change considerably as the statistical model of the source switches between WSCS and WSACS. The resulting analysis enables determining the sampling system parameters in order to facilitate accurate and efficient source coding of acquired CT signals.

## Appendix A. Proof of Lemma 1

**Proof.** To prove that the minimum achievable rate at a given maximum distortion for a code with arbitrary blocklength can be achieved by considering only codes whose blocklength is an integer multiple of *r*, we apply the following approach: We first show that every rate-distortion pair achievable when restricted to using source codes whose blocklength is an integer multiple of *r* is also achievable when using arbitrary blocklenghts; We then prove that every achievable rate-distortion pair is also achievable when restricted to using codes whose blocklength is an integer multiple of *r*. Combining these two assertions proves that the rate-distortion function of the source ${\left\{S\left[i\right]\right\}}_{i\in N}$ can be obtained when restricting the blocklengths to be an integer multiple of *r*. Consequently, a reproduction signal ${\left\{\hat{S}\left[i\right]\right\}}_{i\in N}$ which achieves the minimal rate for a given *D* under the restriction to use only blocklengths which are an integer multiple of *r* is also the reproduction signal achieving the minimal rate without this restriction, and vice versa, thus proving the lemma. To prove the first assertion, consider a rate-distortion pair (R,D) which is achievable when using codes whose blocklength is an integer multiple of *r*. It thus follows directly from Definition 6 that for every η>0, $\exists b_{0}\in N$ such that for all $b>b_{0}$ there exists a a source code $\left(R_{\left(b\cdot r\right)},b\cdot r\right)$ with rate $R_{\left(b\cdot r\right)}\leq R+\eta$ satisfying $\bar{d}\left(S^{\left(b\cdot r\right)},{\hat{S}}^{\left(b\cdot r\right)}\right)\leq D+\frac{\eta }{2}$. We now show that we can construct a code with an arbitrary blocklength l=b·r+j where 0<j<r (i.e., the blocklength *l* is not an integer multiple of *r*) satisfying Definition 6 for all j∈{1,…,r−1} as follows: Apply the code $\left(R_{\left(b\cdot r\right)},b\cdot r\right)$ to the first b·r samples of S[i] and then concatenate each codeword by *j* zeros to obtain a source code having codewords of length b·r+j. The average distortion (i.e., see (2)) of the resulting $\left(R_{\left(b\cdot r+j\right)},b\cdot r+j\right)$ code is given by:

(A1) $$\begin{array}{cc} \bar{d}\left(S^{\left(b\cdot r+j\right)},{\hat{S}}^{\left(b\cdot r+j\right)}\right) & =\frac{1}{b\cdot r+j}\left(\sum_{i=1}^{b\cdot r}E\left\{{\left(S\left[i\right]-\hat{S}\left[i\right]\right)}^{2}\right\}+\sum_{i=b\cdot r+1}^{b\cdot r+j}E\left\{{\left(S[i]\right)}^{2}\right\}\right)\\ \; & =\frac{1}{b\cdot r+j}\left(b\cdot r\cdot \bar{d}\left(S^{\left(b\cdot r\right)},{\hat{S}}^{\left(b\cdot r\right)}\right)+\sum_{i=1}^{j}\sigma_{S}^{2}\left[i\right]\right)\\ \; & =\frac{b\cdot r}{b\cdot r+j}\cdot \bar{d}\left(S^{\left(b\cdot r\right)},{\hat{S}}^{\left(b\cdot r\right)}\right)+\frac{1}{b\cdot r+j}\sum_{i=1}^{j}\sigma_{S}^{2}\left[i\right]. \end{array}$$

Thus, $\exists b_{1}>b_{0}$ such that $\frac{1}{b_{1}\cdot r+j}\sum_{i=1}^{j}\sigma_{S}^{2}\left[i\right]<\frac{\eta }{2}$ and hence, for all $b>b_{1}$

(A2) $$\begin{array}{cc} \bar{d}\left(S^{\left(b\cdot r+j\right)},{\hat{S}}^{\left(b\cdot r+j\right)}\right) & =\frac{b\cdot r}{b\cdot r+j}\cdot \bar{d}\left(S^{\left(b\cdot r\right)},{\hat{S}}^{\left(b\cdot r\right)}\right)+\frac{1}{b\cdot r+j}\sum_{i=1}^{j}\sigma_{S}^{2}\left[i\right]\\ \; & \leq \frac{b\cdot r}{b\cdot r+j}\cdot \bar{d}\left(S^{\left(b\cdot r\right)},{\hat{S}}^{\left(b\cdot r\right)}\right)+\frac{\eta }{2}\\ \; & \leq \bar{d}\left(S^{\left(b\cdot r\right)},{\hat{S}}^{\left(b\cdot r\right)}\right)+\frac{\eta }{2}\leq D+\eta . \end{array}$$

The rate $R_{\left(b\cdot r+j\right)}$ satisfies:

(A3) $$R_{\left(b\cdot r+j\right)}=\frac{1}{b\cdot r+j}\cdot \log_{2}M=R_{\left(b\cdot r\right)}\cdot \frac{b\cdot r}{b\cdot r+j}\leq \left(R+\eta \right)\cdot \frac{b\cdot r}{b\cdot r+j}\leq R+\eta .$$

Consequently, any rate-distortion pair achievable with codes whose blocklength is an integer multiple of *r* can be achieved by codes with arbitrary blocklengths. Next, we prove that any achievable rate-distortion pair (R,D) can be achieved by codes whose blocklength is an integer multiple of *r*. To that aim, we fix η>0. By Definition 6, it holds that there exists a code of blocklength *l* satisfying (3) and (4). To show that (R,D) is achievable using codes whose blocklength is an integer multiple of *r*, we assume here that *l* is not an integer multiple of *r*, hence, there exist some positive integers *b* and *j* such that j<r and l=b·r+j. We denote this code by $\left(R_{\left(b\cdot r+j\right)},b\cdot r+j\right)$. It follows from Definition 6 that $R_{\left(b\cdot r+j\right)}\leq R+\eta$ and $\bar{d}\left(S^{\left(b\cdot r+j\right)},{\hat{S}}^{\left(b\cdot r+j\right)}\right)\leq D+\frac{\eta }{2}$. Next, we construct a code $\left(R_{(b+1)\cdot r},\left(b+1\right)\cdot r\right)$ with codewords whose length is (b+1)·r, i.e., an integer multiple of *r*, by adding r−j zeros at the end of each codeword of the code $\left(R_{\left(b\cdot r+j\right)},b\cdot r+j\right)$. The average distortion can now be computed as follows:

(A4) $$\begin{array}{cc} \bar{d}\left(S^{\left((b+1)\cdot r\right)},{\hat{S}}^{\left((b+1)\cdot r\right)}\right) & =\frac{1}{(b+1)\cdot r}\left(\sum_{i=1}^{b\cdot r+j}E\left\{{\left(S\left[i\right]-\hat{S}\left[i\right]\right)}^{2}\right\}+\sum_{i=b\cdot r+j+1}^{(b+1)\cdot r}E\left\{{\left(S[i]\right)}^{2}\right\}\right)\\ \; & =\frac{1}{(b+1)\cdot r}\left(\left(b\cdot r+j\right)\cdot \bar{d}\left(S^{\left(b\cdot r+j\right)},{\hat{S}}^{\left(b\cdot r+j\right)}\right)+\sum_{i=b\cdot r+j+1}^{(b+1)\cdot r}\sigma_{S}^{2}\left[i\right]\right)\\ \; & =\frac{b\cdot r+j}{(b+1)\cdot r}\cdot \bar{d}\left(S^{\left(b\cdot r+j\right)},{\hat{S}}^{\left(b\cdot r+j\right)}\right)+\frac{\sum_{i=b\cdot r+j+1}^{(b+1)\cdot r}\sigma_{S}^{2}\left[i\right]}{(b+1)\cdot r}, \end{array}$$

Thus, since the variance is finite and bounded, $\exists b_{1}>b_{0}$ such that $\frac{\sum_{i=b_{1}\cdot r+j+1}^{(b_{1}+1)\cdot r}\sigma_{S}^{2}\left[i\right]}{(b_{1}+1)\cdot r}<\frac{\eta }{2}$ for all $b>b_{1}$. Hence, for all $b>b_{1}$

(A5) $$\begin{array}{cc} \bar{d}\left(S^{\left((b+1)\cdot r\right)},{\hat{S}}^{((b+1)\cdot r}\right) & \leq \frac{b\cdot r+j}{(b+1)\cdot r}\cdot \bar{d}\left(S^{\left(b\cdot r+j\right)},{\hat{S}}^{\left(b\cdot r+j\right)}\right)+\frac{\eta }{2}\\ \; & \leq \bar{d}\left(S^{\left(b\cdot r+j\right)},{\hat{S}}^{\left(b\cdot r+j\right)}\right)+\frac{\eta }{2}\leq D+\eta . \end{array}$$

The rate $R_{(b+1)\cdot r}$ can be expressed as follows:

(A6) $$R_{(b+1)\cdot r}=\frac{1}{(b+1)\cdot r}\cdot \log_{2}M=R_{\left(b\cdot r+j\right)}\cdot \frac{b\cdot r+j}{(b+1)\cdot r}\leq \left(R+\eta \right)\cdot \frac{b\cdot r+j}{(b+1)\cdot r}<R+\eta .$$

It follows that $R_{(b+1)\cdot r}\leq R+\eta$ for any arbitrary η by selecting a sufficiently large *b*. This proves that every rate-distortion pair achievable with arbitrary blocklengths (e.g., l=b·r+j,j<r) is also achievable when considering source codes whose blocklength is an integer multiple of *r* (i.e., l=b·r). This concludes the proof. □

## Appendix B. Proof of Theorem 1

Recall that $\alpha \in R^{++}$. To prove the theorem, we fix a rate-distortion pair (R,D) that is achievable for the source ${\left\{S\left[i\right]\right\}}_{i\in N}$. By Definition 6 this implies that for all η>0 there exists $l_{0}\left(\eta \right)\in N$ such that for all $l>l_{0}\left(\eta \right)$ there exists a source code $C_{l}$ with rate $R_{\left(l\right)}\leq R+\eta$ and MSE distortion $D_{\left(l\right)}=E\left\{\frac{1}{l}∥S^{\left(l\right)}-{\hat{S}}^{\left(l\right)}{∥}^{2}\right\}\leq D+\eta$, where ∥·∥ denotes the norm of a vector. Next, we use the code $C_{l}$ to define the source code $C_{l}^{\left(\alpha \right)}$, which operates in the following manner: The encoder first scales its input block by 1/α. Then, the block is encoded using the source code $C_{l}$. Finally, the selected codeword is scaled by α. Since the $C_{l}^{\left(\alpha \right)}$ has the same number of codewords and the same blocklength as $C_{l}$, it follows that its rate, denote $R_{\left(l\right)}^{\left(\alpha \right)}$, satisfied $R_{\left(l\right)}^{\left(\alpha \right)}=R_{\left(l\right)}\leq R+\eta$. Furthermore, by the construction of $C_{l}^{\left(\alpha \right)}$, it holds that its reproduction vector when applied to $\alpha \cdot S^{\left(l\right)}$ is equal to the output of $C_{l}$ applied to $S^{\left(l\right)}$ scaled by α, i.e., $\alpha \cdot {\hat{S}}^{\left(l\right)}$. Consequently, the MSE of $C_{l}^{\left(\alpha \right)}$ when applied to the source ${\left\{\alpha \cdot S\left[i\right]\right\}}_{i\in N}$, denoted $D_{\left(l\right)}^{\left(\alpha \right)}$, satisfies $D_{\left(l\right)}^{\left(\alpha \right)}=E\left\{\frac{1}{l}∥\alpha \cdot S^{\left(l\right)}-\alpha \cdot {\hat{S}}^{\left(l\right)}{∥}^{2}\right\}=\alpha^{2}\cdot D_{\left(l\right)}\leq \alpha^{2}\cdot D+\alpha^{2}\eta$.

It thus follows that for all $\tilde{\eta }>0$ there exists ${\tilde{l}}_{0}\left(\tilde{\eta }\right)=l_{0}\left(\min(\tilde{\eta },\alpha^{2}\tilde{\eta })\right)$ such that for all $l>{\tilde{l}}_{0}\left(\tilde{\eta }\right)$ there exists a code $C_{l}^{\left(\alpha \right)}$ with rate $R_{\left(l\right)}^{\left(\alpha \right)}\leq R+\tilde{\eta }$ which achieves an MSE distortion of $D_{\left(l\right)}^{\left(\alpha \right)}\leq \alpha^{2}\cdot D+\tilde{\eta }$ when applied to the compression of ${\left\{\alpha \cdot S\left[i\right]\right\}}_{i\in N}$. Hence, $\left(R,\alpha^{2}\cdot D\right)$ is achievable for compression of ${\left\{\alpha \cdot S\left[i\right]\right\}}_{i\in N}$ by Definition 6, proving the theorem.

## Appendix C. Proof of Theorem 3

In this appendix, we prove (17b) by applying a similar approach as used for proving (17a) in ([17] Appendix A). We first note that Definition 9 can also be written as follows:

(A7) $$p-\underset{k\to \infty }{\lim\sup}Z_{k}\;\overset{\left(a\right)}{=}\;\inf\left\{\beta \in R|\underset{k\to \infty }{\lim\sup}\mathrm{Pr}\left(Z_{k}>\beta \right)=0\right\}\;\overset{\left(b\right)}{=}\;\inf\left\{\beta \in R|\underset{k\to \infty }{\lim\inf}F_{k}\left(\beta \right)=1\right\}.$$

For the equality (a), we note that the set of probabilities ${\left\{\mathrm{Pr}\left(Z_{k}>\beta \right)\right\}}_{k\in N}$ is non-negative and bounded in [0,1]; hence, for any β∈R for which $\underset{k\to \infty }{\lim\sup}\mathrm{Pr}\left(Z_{k}>\beta \right)=0$, it also holds from ([31] Theorem 3.17) that the limit of any subsequence of ${\left\{\mathrm{Pr}\left(Z_{k}>\beta \right)\right\}}_{k\in N}$ is also 0, since non-negativity of the probability implies $\underset{k\to \infty }{\lim\inf}\mathrm{Pr}\left(Z_{k}>\beta \right)\geq 0$. Then, combined with the relationship $\underset{k\to \infty }{\lim\inf}\mathrm{Pr}\left(Z_{k}>\beta \right)\leq \underset{k\to \infty }{\lim\sup}\mathrm{Pr}\left(Z_{k}>\beta \right)$, we conclude:

$$\begin{array}{c} 0\leq \underset{k\to \infty }{\lim\inf}\mathrm{Pr}\left(Z_{k}>\beta \right)\leq \underset{k\to \infty }{\lim\sup}\mathrm{Pr}\left(Z_{k}>\beta \right)=0\\ \;\Rightarrow \underset{k\to \infty }{\lim\inf}\mathrm{Pr}\left(Z_{k}>\beta \right)=\underset{k\to \infty }{\lim\sup}\mathrm{Pr}\left(Z_{k}>\beta \right)\overset{\left(a\right)}{=}\lim_{k\to \infty }\mathrm{Pr}\left(Z_{k}>\beta \right)=0, \end{array}$$

where (a) follows from ([31] Example 3.18(c)). This implies $\lim_{k\to \infty }\mathrm{Pr}\left(Z_{k}>\beta \right)$ exists and is equal to 0.

In the opposite direction, if $\lim_{k\to \infty }\mathrm{Pr}\left(Z_{k}>\beta \right)=0$ then it follows from ([31] Example 3.18(c)) that $\underset{k\to \infty }{\lim\sup}\mathrm{Pr}\left(Z_{k}>\beta \right)=0$. Next, we note that since $F_{k}\left(\beta \right)$ is bounded in [0,1] then $\underset{k\to \infty }{\lim\inf}F_{k}\left(\beta \right)$ is finite ∀β∈R, even if $\lim_{k\to \infty }F_{k}\left(\beta \right)$ does not exist. Equality (b) follows since $\underset{k\to \infty }{\lim\sup}\mathrm{Pr}\left(Z_{k}>\beta \right)=\underset{k\to \infty }{\lim\sup}\left(1-\mathrm{Pr}\left(Z_{k}\leq \beta \right)\right)$ which according to ([32] Theorem 7.3.7) is equal to $1+\underset{k\to \infty }{\lim\sup}\left(-\mathrm{Pr}\left(Z_{k}\leq \beta \right)\right)$. By ([33] Chapter 1, page 29), this quantity is also equal to $1-\underset{k\to \infty }{\lim\inf}\left(\mathrm{Pr}\left(Z_{k}\leq \beta \right)\right)=1-\underset{k\to \infty }{\lim\inf}F_{k}\left(\beta \right)$.

Next, we state the following lemma:

**Lemma** **A1.**
*Given assumption AS2, for all* β∈R *it holds that*

(A8) $$\underset{k\to \infty }{\lim\inf}F_{k}\left(\beta \right)=\lim_{n\to \infty }\underset{k\to \infty }{\lim\inf}{\tilde{F}}_{k,n}\left(\beta \right).$$

**Proof.** To prove the lemma we first show that $\underset{k\to \infty }{\lim\inf}F_{k}\left(\beta \right)\leq \lim_{n\to \infty }\underset{k\to \infty }{\lim\inf}{\tilde{F}}_{k,n}\left(\beta \right)$, and then we show $\underset{k\to \infty }{\lim\inf}F_{k}\left(\beta \right)\geq \lim_{n\to \infty }\underset{k\to \infty }{\lim\inf}{\tilde{F}}_{k,n}\left(\beta \right)$. Recall that by AS2, for all β∈R and k∈N, ${\tilde{F}}_{k,n}\left(\beta \right)$ converges as n→∞ to $F_{k}\left(\beta \right)$, uniformly over *k* and β, i.e., for all η>0 there exists $n_{0}\left(\eta \right)\in N$, $k_{0}\left(n_{0}\left(\eta \right),\eta \right)\in N$ such that for every $n>n_{0}\left(\eta \right)$, β∈R and $k>k_{0}\left(n_{0}\left(\eta \right),\eta \right)$, it holds that $|{\tilde{F}}_{k,n}\left(\beta \right)-F_{k}\left(\beta \right)|<\eta$. Consequently, for every subsequence $0<k_{1}<k_{2}<\ldots$ such that $\lim_{l\to \infty }{\tilde{F}}_{k_{l},n}\left(\beta \right)$ exists for any $n>n_{0}\left(\eta \right)$, it follows from ([31] Theorem 7.11) that, as the convergence over *k* is uniform, the limits over *n* and *l* are interchangeable:

(A9) $$\lim_{n\to \infty }\lim_{l\to \infty }{\tilde{F}}_{k_{l},n}\left(\beta \right)=\lim_{l\to \infty }\lim_{n\to \infty }{\tilde{F}}_{k_{l},n}\left(\beta \right)=\lim_{l\to \infty }F_{k_{l}}\left(\beta \right).$$

The existence of such a convergent subsequence is guaranteed by the Bolzano-Weierstrass theorem ([31] Theorem 2.42) as ${\tilde{F}}_{k,n}\left(\beta \right)\in \left[0,1\right]$. From the properties of the limit inferior ([31] Theorem 3.17) it follows that there exists a subsequence of ${\left\{F_{k}\left(\beta \right)\right\}}_{k\in N}$, denoted ${\left\{F_{k_{m}}\left(\beta \right)\right\}}_{m\in N}$, such that $\lim_{m\to \infty }F_{k_{m}}\left(\beta \right)=\underset{k\to \infty }{\lim\inf}F_{k}\left(\beta \right)$. Consequently,

(A10) $$\begin{array}{cc} \underset{k\to \infty }{\lim\inf}F_{k}\left(\beta \right) & =\lim_{m\to \infty }F_{k_{m}}\left(\beta \right)\overset{\left(a\right)}{=}\lim_{n\to \infty }\lim_{m\to \infty }{\tilde{F}}_{k_{m},n}\left(\beta \right)\\ \; & \overset{\left(b\right)}{\geq }\lim_{n\to \infty }\underset{k\to \infty }{\lim\inf}{\tilde{F}}_{k,n}\left(\beta \right), \end{array}$$

where (a) follows from (A9), and (b) follows from the definition of the limit inferior ([31] Definition 3.16). Similarly, by ([31] Theorem 3.17), for any n∈N there exists a subsequence of ${\left\{{\tilde{F}}_{k,n}\left(\beta \right)\right\}}_{k\in N}$ which we denote by ${\left\{{\tilde{F}}_{k_{l},n}\left(\beta \right)\right\}}_{l\in N}$ where ${\left\{k_{l}\right\}}_{l\in N}$ satisfy $0<k_{1}<k_{2}<\ldots$, such that $\lim_{l\to \infty }{\tilde{F}}_{k_{l},n}\left(\beta \right)=\underset{k\to \infty }{\lim\inf}{\tilde{F}}_{k,n}\left(\beta \right)$. Therefore,

(A11) $$\begin{array}{cc} \lim_{n\to \infty }\underset{k\to \infty }{\lim\inf}{\tilde{F}}_{k,n}\left(\beta \right) & =\lim_{n\to \infty }\lim_{l\to \infty }{\tilde{F}}_{k_{l},n}\left(\beta \right)\\ \; & \overset{\left(a\right)}{=}\lim_{l\to \infty }F_{k_{l}}\left(\beta \right)\overset{\left(b\right)}{\geq }\underset{k\to \infty }{\lim\inf}F_{k}\left(\beta \right), \end{array}$$

where (a) follows from (A9), and (b) follows from the definition of the limit inferior ([31] Definition 3.16). Therefore, $\underset{k\to \infty }{\lim\inf}F_{k}\left(\beta \right)\leq \lim_{n\to \infty }\underset{k\to \infty }{\lim\inf}{\tilde{F}}_{k,n}\left(\beta \right)$. Combining (A10) and (A11) proves (A8) in the statement of the lemma. □

**Lemma** **A2.**
*Given assumptions AS1–AS2, the sequence of RVs* ${\left\{{\tilde{Z}}_{k,n}\right\}}_{k,n\in N}$ *satisfies*

(A12) $$\begin{array}{cc} \lim_{n\to \infty }\left(p-\underset{k\to \infty }{\lim\sup}{\tilde{Z}}_{k,n}\right) & =\inf\left\{\beta \in R|\lim_{n\to \infty }\underset{k\to \infty }{\lim\inf}{\tilde{F}}_{k,n}\left(\beta \right)=1\right\}. \end{array}$$

**Proof.** Since by assumption AS1, for every n∈N, every convergent subsequence of ${\left\{{\tilde{Z}}_{k,n}\right\}}_{k\in N}$ converges in distribution as k→∞ to a deterministic scalar, it follows that every convergent subsequence of ${\tilde{F}}_{k,n}\left(\beta \right)$ converges as k→∞ to a step function, which is the CDF of the corresponding sublimit of ${\tilde{Z}}_{k,n}$. In particular, the limit $\underset{k\to \infty }{\lim\inf}{\tilde{F}}_{k,n}\left(\beta \right)$ is a step function representing the CDF of the deterministic scalar $\zeta_{n}$, i.e.,

(A13) $$\underset{k\to \infty }{\lim\inf}{\tilde{F}}_{k,n}\left(\beta \right)=\left\{\begin{array}{cc} 0 & \beta <\zeta_{n}\\ 1 & \beta \geq \zeta_{n}. \end{array}\right.$$

Since, by Lemma A1, AS2 implies that the limit $\lim_{n\to \infty }\underset{k\to \infty }{\lim\inf}{\tilde{F}}_{k,n}\left(\beta \right)$ exists (convergence to a discontinuous function is in the sense of ([31] Ex. 7.3)), then $\lim_{n\to \infty }\zeta_{n}$ exists. Hence, we obtain that

(A14) $$\lim_{n\to \infty }\underset{k\to \infty }{\lim\inf}{\tilde{F}}_{k,n}\left(\beta \right)=\left\{\begin{array}{cc} 0 & \beta <\lim_{n\to \infty }\zeta_{n}\\ 1 & \beta \geq \lim_{n\to \infty }\zeta_{n}, \end{array}\right.$$

and from the right-hand side of (A12) we have that

(A15) $$\inf\left\{\beta \in R|\lim_{n\to \infty }\underset{k\to \infty }{\lim\inf}{\tilde{F}}_{k,n}\left(\beta \right)=1\right\}=\lim_{n\to \infty }\zeta_{n}.$$

Next, from (A7) and (A13) we note that

$$\begin{array}{c} p-\underset{k\to \infty }{\lim\sup}{\tilde{Z}}_{k,n}=\inf\left\{\beta \in R|\underset{k\to \infty }{\lim\inf}{\tilde{F}}_{k,n}\left(\beta \right)=1\right\}=\zeta_{n}. \end{array}$$

Consequently, the left-hand side of (A12) is equal to $\lim_{n\to \infty }\zeta_{n}$. Combining with (A15) we arrive at the equality (A12) in the statement of the lemma. □

Substituting (A8) into (A7) results in

(A16) $$\begin{array}{cc} p-\underset{k\to \infty }{\lim\sup}Z_{k}\; & =\inf\left\{\beta \in R|\lim_{n\to \infty }\underset{k\to \infty }{\lim\inf}{\tilde{F}}_{k,n}\left(\beta \right)=1\right\}\overset{\left(a\right)}{=}\lim_{n\to \infty }\left(p-\underset{k\to \infty }{\lim\sup}{\tilde{Z}}_{k,n}\right), \end{array}$$

where (a) follows from (A12). Equation (A16) concludes the proof for (17b).

## Appendix D. Proof of Theorem 4

In this appendix we detail the proof of Theorem 4. The outline of the proof is given as follows:

- We first show in Appendix D.1 that for any k∈N, the PDF of the random vector $S_{n}^{\left(k\right)}$, representing the first *k* samples of the CT WSCS source $S_{c}\left(t\right)$ sampled at time instants $T_{s}\left(n\right)=\frac{T_{\mathrm{ps}}}{p+\epsilon_{n}}$, converges in the limit as n→∞ and for any k∈N to the PDF of $S_{\epsilon }^{\left(k\right)}$, which represents the first *k* samples of the CT WSCS source $S_{c}\left(t\right)$, sampled at time instants $T_{s}=\frac{T_{\mathrm{ps}}}{p+\epsilon }$. We prove that this convergence is uniform in k∈N and in the realization vector $s^{\left(k\right)}\in R^{k}$. This is stated in Lemma A3.
- Next, in Appendix D.2 we apply Theorem 3 to relate the mutual information density rates for the random source vector $S_{n}^{\left(k\right)}$ and its reproduction ${\hat{S}}_{n}^{\left(k\right)}$ with that of the random source vector $S_{\epsilon }^{\left(k\right)}$ and its reproduction ${\hat{S}}_{\epsilon }^{\left(k\right)}$. To that aim, let the functions $F_{S_{n},{\hat{S}}_{n}}$ and $F_{S_{\epsilon },{\hat{S}}_{\epsilon }}$ denote the joint distributions of an arbitrary dimensional source and reproduction vectors corresponding to the synchronously sampled and to the asynchronously sampled source process respectively. We define the following mutual information density rates:
  (A17a) $${\tilde{Z}}_{k,n}^{\prime }\left(F_{S_{n},{\hat{S}}_{n}}\right)≜\frac{1}{k}\log\frac{p_{S_{n}^{\left(k\right)}|{\hat{S}}_{n}^{\left(k\right)}}\left(S_{n}^{\left(k\right)}|{\hat{S}}_{n}^{\left(k\right)}\right)}{p_{S_{n}^{\left(k\right)}}\left(S_{n}^{\left(k\right)}\right)},$$
  and
  (A17b) $$Z_{k,\epsilon }^{\prime }\left(F_{S_{\epsilon },{\hat{S}}_{\epsilon }}\right)≜\frac{1}{k}\log\frac{p_{S_{\epsilon }^{\left(k\right)}|{\hat{S}}_{\epsilon }^{\left(k\right)}}\left(S_{\epsilon }^{\left(k\right)}|{\hat{S}}_{\epsilon }^{\left(k\right)}\right)}{p_{S_{\epsilon }^{\left(k\right)}}\left(S_{\epsilon }^{\left(k\right)}\right)},$$
  k,n∈N. The RVs ${\tilde{Z}}_{k,n}^{\prime }\left(F_{S_{n},{\hat{S}}_{n}}\right)$ and $Z_{k,\epsilon }^{\prime }\left(F_{S_{\epsilon },{\hat{S}}_{\epsilon }}\right)$ in (A17) denote the mutual information density rates ([14] Definition 3.2.1) between the DT source process and the corresponding reproduction process for the case of synchronous sampling and for the case of asynchronous sampling, respectively.
  We then show that if the pairs of source process and optimal reproduction process ${\left\{S_{n}\left[i\right],{\hat{S}}_{n}\left[i\right]\right\}}_{i\in N}$ and ${\left\{S_{\epsilon }\left[i\right],{\hat{S}}_{\epsilon }\left[i\right]\right\}}_{i\in N}$ satisfy that $p_{{\hat{S}}_{n}^{\left(k\right)}}\left({\hat{s}}^{\left(k\right)}\right)\underset{n\to \infty }{⟶}p_{{\hat{S}}_{\epsilon }^{\left(k\right)}}\left({\hat{s}}^{\left(k\right)}\right)$ uniformly with respect to ${\hat{s}}^{\left(k\right)}\in R^{k}$ and k∈N, and that $p_{S_{n}^{\left(k\right)}|{\hat{S}}_{n}^{\left(k\right)}}\left(s^{\left(k\right)}|{\hat{s}}^{\left(k\right)}\right)\underset{n\to \infty }{⟶}p_{S_{\epsilon }^{\left(k\right)}|{\hat{S}}_{\epsilon }^{\left(k\right)}}\left(s^{\left(k\right)}|{\hat{s}}^{\left(k\right)}\right)$ uniformly in ${\left({\left({\hat{s}}^{\left(k\right)}\right)}^{T},{\left(s^{\left(k\right)}\right)}^{T}\right)}^{T}\in R^{2k}$ and k∈N, then ${\tilde{Z}}_{k,n}^{\prime }\left(F_{S_{n},{\hat{S}}_{n}}\right)\overset{\left(dist.\right)}{\underset{n\to \infty }{⟶}}Z_{k,\epsilon }^{\prime }\left(F_{S_{\epsilon },{\hat{S}}_{\epsilon }}\right)$ uniformly in k∈N. In addition, Lemma A5 proves that every subsequence of ${\left\{{\tilde{Z}}_{k,n}^{\prime }\left(F_{S_{n},{\hat{S}}_{n}}\right)\right\}}_{k\in N}$w.r.t.*k*, indexed as $k_{l}$ converges in distribution, in the limit l→∞ to a deterministic scalar.
- Lastly, in Appendix D.3 we combine the above results to show in Lemmas A7 and A8 that $R_{\epsilon }\left(D\right)\leq \underset{n\to \infty }{\lim\sup}R_{n}\left(D\right)$ and $R_{\epsilon }\left(D\right)\geq \underset{n\to \infty }{\lim\sup}R_{n}\left(D\right)$ respectively; implying that $R_{\epsilon }\left(D\right)=\underset{n\to \infty }{\lim\sup}R_{n}\left(D\right)$, which proves the theorem.

To facilitate our proof we will need uniform convergence in k∈N, of $p_{S_{n}^{\left(k\right)}}\left(s^{\left(k\right)}\right)$, $p_{{\hat{S}}_{n}^{\left(k\right)}}\left({\hat{s}}^{\left(k\right)}\right)$ and $p_{S_{n}^{\left(k\right)}|{\hat{S}}_{n}^{\left(k\right)}}\left(s^{\left(k\right)}|{\hat{s}}^{\left(k\right)}\right)$ to $p_{S_{\epsilon }^{\left(k\right)}}\left(s^{\left(k\right)}\right)$, $p_{{\hat{S}}_{\epsilon }^{\left(k\right)}}\left({\hat{s}}^{\left(k\right)}\right)$ and $p_{S_{\epsilon }^{\left(k\right)}|{\hat{S}}_{\epsilon }^{\left(k\right)}}\left(s^{\left(k\right)}|{\hat{s}}^{\left(k\right)}\right)$, respectively. To that aim, we will make the following scaling assumption w.l.o.g.:

**Assumption** **A1.**
*The variance of the source and the allowed distortion are scaled by some factor* $\alpha^{2}$ *such that*

(A18) $$\alpha^{2}\cdot \min\left\{D,\left(\min_{0\leq t\leq T_{\mathrm{ps}}}\sigma_{S_{c}}^{2}\left(t\right)-D\right)\right\}>\frac{1}{2\pi }.$$

Note that this assumption has no effect on the generality of the RDF for multivariate stationary processes detailed in ([5] Section 10.3.3), ([34] Section IV). Moreover, by Theorem 1, for every α>0 it holds that when any rate *R* achievable when compressing the original source $S_{c}\left(t\right)$ with distortion not larger that *D* is achievable when compressing the scaled source $\alpha \cdot S_{c}\left(t\right)$ with distortion not larger than $\alpha^{2}\cdot D$. Note that if for the source $S_{c}\left(t\right)$ the distortion satisfies $D<\min_{0\leq t\leq T_{\mathrm{ps}}}\sigma_{S_{c}}^{2}\left(t\right)$, then for the scaled source and distortion we have $\alpha^{2}\cdot D<\min_{0\leq t\leq T_{\mathrm{ps}}}\alpha^{2}\cdot \sigma_{S_{c}}^{2}\left(t\right)$.

### Appendix D.1. Convergence in Distribution of $S_{n}^{\left(k\right)}$ to $S_{\epsilon }^{\left(k\right)}$ Uniformly with Respect to k∈N

In order to prove the uniform convergence in distribution, $S_{n}^{\left(k\right)}\overset{\left(dist.\right)}{\underset{n\to \infty }{⟶}}S_{\epsilon }^{\left(k\right)}$, uniformly with respect to k∈N, we first prove, in Lemma A3, that as n→∞ the sequence of PDFs of $S_{n}^{\left(k\right)}$, $p_{S_{n}^{\left(k\right)}}\left(s^{\left(k\right)}\right)$, converges to the PDF of $S_{\epsilon }^{\left(k\right)}$, $p_{S_{\epsilon }^{\left(k\right)}}\left(s^{\left(k\right)}\right)$, uniformly in $s^{\left(k\right)}\in R^{k}$ and in k∈N. Next, we show in Corollary A1 that $S_{n}^{\left(k\right)}\overset{\left(dist.\right)}{\underset{n\to \infty }{⟶}}S_{\epsilon }^{\left(k\right)}$ uniformly in k∈N.

To that aim, let us define the set K≜{1,2,…,k} and consider the *k*- dimensional zero-mean, memoryless random vectors $S_{n}^{\left(k\right)}$ and $S_{\epsilon }^{\left(k\right)}$ with their respective diagonal correlation matrices expressed below:

(A19a) $$R_{n}^{\left(k\right)}≜E\left\{\left(S_{n}^{\left(k\right)}\right){\left(S_{n}^{\left(k\right)}\right)}^{T}\right\}=\mathrm{diag}\left(\sigma_{S_{n}}^{2}\left[1\right],\ldots ,\sigma_{S_{n}}^{2}\left[k\right]\right),$$

(A19b) $$R_{\epsilon }^{\left(k\right)}≜E\left\{\left(S_{\epsilon }^{\left(k\right)}\right){\left(S_{\epsilon }^{\left(k\right)}\right)}^{T}\right\}=\mathrm{diag}\left(\sigma_{S_{\epsilon }}^{2}\left[1\right],\ldots ,\sigma_{S_{\epsilon }}^{2}\left[k\right]\right).$$

Since $\epsilon_{n}≜\frac{\left\lfloor n\cdot \epsilon \right\rfloor }{n}$ it holds that $\frac{n\cdot \epsilon -1}{n}\leq \epsilon_{n}\leq \frac{n\cdot \epsilon }{n}$; therefore

(A20) $$\lim_{n\to \infty }\epsilon_{n}=\epsilon .$$

Now we note that since $\sigma_{S_{c}}^{2}\left(t\right)$ is uniformly continuous, then by the definition of a uniformly continuous function, for each i∈N, the limit in (A20) implies that

(A21) $$\lim_{n\to \infty }\sigma_{S_{n}}^{2}\left[i\right]\equiv \lim_{n\to \infty }\sigma_{S_{c}}^{2}\left(i\cdot \frac{T_{\mathrm{ps}}}{p+\epsilon_{n}}\right)=\sigma_{S_{c}}^{2}\left(i\cdot \frac{T_{\mathrm{ps}}}{p+\epsilon }\right)\equiv \sigma_{S_{\epsilon }}^{2}\left[i\right].$$

From Assumption A1, it follows that $\sigma_{S_{n}}^{2}\left[i\right]$ satisfies $\sigma_{S_{n}}^{2}\left[i\right]>\frac{1}{2\pi }$; Hence, we can state the following lemma:

**Lemma** **A3.**
*The PDF of* $S_{n}^{\left(k\right)}$ *,* $p_{S_{n}^{\left(k\right)}}\left(s^{\left(k\right)}\right)$ *, converges as* n→∞ *to the PDF of* $S_{\epsilon }^{\left(k\right)}$ *,* $p_{S_{\epsilon }^{\left(k\right)}}\left(s^{\left(k\right)}\right)$ *, uniformly in* $s^{\left(k\right)}\in R^{k}$ *and in* k∈N *:*

$$\lim_{n\to \infty }p_{S_{n}^{\left(k\right)}}\left(s^{\left(k\right)}\right)=p_{S_{\epsilon }^{\left(k\right)}}\left(s^{\left(k\right)}\right),\;\forall s^{\left(k\right)}\in R^{k},\forall k\in N.$$

**Proof.** The proof of the lemma directly follows from the steps in the proof of ([17] Lemma B.1), which was applied to random Gaussian processes with independent entries and variance larger than $\frac{1}{2\pi }$. □

Lemma A3 gives rise to the following corollary:

**Corollary** **A1.**
*For any* k∈N *it holds that* $S_{n}^{\left(k\right)}\overset{\left(dist.\right)}{\underset{n\to \infty }{⟶}}S_{\epsilon }^{\left(k\right)}$ *, and convergence is uniform over k.*

**Proof.** The corollary holds due to ([35] Theorem 1): Since $p_{S_{n}^{\left(k\right)}}\left(s^{\left(k\right)}\right)$ converges to $p_{S_{\epsilon }^{\left(k\right)}}\left(s^{\left(k\right)}\right)$ then $S_{n}^{\left(k\right)}\overset{\left(dist.\right)}{\underset{n\to \infty }{⟶}}S_{\epsilon }^{\left(k\right)}$. In addition, since the convergence of the PDFs is uniform in k∈N, the convergence of the CDFs is also uniform in k∈N. □

### Appendix D.2. Showing that ${\tilde{Z}}_{k,n}^{\prime }\left(F_{S_{n},{\hat{S}}_{n}}^{\mathrm{opt}}\right)$ and $Z_{k,\epsilon }^{\prime }\left(F_{S_{\epsilon },{\hat{S}}_{\epsilon }}\right)$ Satisfy the Conditions of Theorem 3

Let $F_{S_{n},{\hat{S}}_{n}}^{\mathrm{opt}}$ denote the joint distribution for the source process and the corresponding optimal reproduction process satisfying the distortion constraint *D*. We next prove that for $F_{S_{n},{\hat{S}}_{n}}^{\mathrm{opt}}\overset{\left(dist.\right)}{\underset{n\to \infty }{⟶}}F_{S_{\epsilon },{\hat{S}}_{\epsilon }}$, then ${\tilde{Z}}_{k,n}^{\prime }\left(F_{S_{n},{\hat{S}}_{n}}^{\mathrm{opt}}\right)$ and $Z_{k,\epsilon }^{\prime }\left(F_{S_{\epsilon },{\hat{S}}_{\epsilon }}\right)$ satisfy AS1–AS2. In particular, Lemma A4 proves that ${\tilde{Z}}_{k,n}^{\prime }\left(F_{S_{n},{\hat{S}}_{n}}^{\mathrm{opt}}\right)\overset{\left(dist.\right)}{\underset{n\to \infty }{⟶}}Z_{k,\epsilon }^{\prime }\left(F_{S_{\epsilon },{\hat{S}}_{\epsilon }}\right)$ uniformly in k∈N for the optimal zero-mean Gaussian reproduction vectors with independent entries. Lemma A5 proves that for any fixed *n*, ${\tilde{Z}}_{k,n}^{\prime }\left(F_{S_{n},{\hat{S}}_{n}}^{\mathrm{opt}}\right)$ converges in distribution to a deterministic scalar as k→∞.

**Lemma** **A4.**
*Let* ${\left\{{\hat{S}}_{n}^{\left(k\right)}\right\}}_{n\in N}$ *and* ${\left\{W_{n}^{\left(k\right)}\right\}}_{n\in N}$ *be two sets of mutually independent sequences of* k×1 *zero-mean Gaussian random vectors related via the backward channel *(20)*, each having independent entries and let PDFs* $p_{{\hat{S}}_{n}^{\left(k\right)}}\left({\hat{s}}^{\left(k\right)}\right)$ *and* $p_{W_{n}^{\left(k\right)}}\left(w^{\left(k\right)}\right)$ *, respectively, denote their PDFs. Consider two other zero-mean Gaussian random vectors* ${\hat{S}}_{\epsilon }^{\left(k\right)}$ *and* $W_{\epsilon }^{\left(k\right)}$ *each having independent entries with the PDFs* $p_{{\hat{S}}_{\epsilon }^{\left(k\right)}}\left({\hat{s}}^{\left(k\right)}\right)$ *and* $p_{W_{\epsilon }^{\left(k\right)}}\left(w^{\left(k\right)}\right)$ *, respectively, such that* $\lim_{n\to \infty }p_{{\hat{S}}_{n}^{\left(k\right)}}\left({\hat{s}}^{\left(k\right)}\right)=p_{{\hat{S}}_{\epsilon }^{\left(k\right)}}\left({\hat{s}}^{\left(k\right)}\right)$ *uniformly in* ${\hat{s}}^{\left(k\right)}\in R^{k}$ *and uniformly with respect to* k∈N *, and* $\lim_{n\to \infty }p_{W_{n}^{\left(k\right)}}\left(w^{\left(k\right)}\right)=p_{W_{\epsilon }^{\left(k\right)}}\left(w^{\left(k\right)}\right)$ *uniformly in* $w^{\left(k\right)}\in R^{k}$ *and uniformly with respect to* k∈N *. Then, the RVs* ${\tilde{Z}}_{k,n}^{\prime }\left(F_{S_{n},{\hat{S}}_{n}}^{\mathrm{opt}}\right)$ *and* $Z_{k,\epsilon }^{\prime }\left(F_{S_{\epsilon },{\hat{S}}_{\epsilon }}\right)$ *, defined via *(A17)* satisfy* ${\tilde{Z}}_{k,n}^{\prime }\left(F_{S_{n},{\hat{S}}_{n}}^{\mathrm{opt}}\right)\overset{\left(dist.\right)}{\underset{n\to \infty }{⟶}}Z_{k,\epsilon }^{\prime }\left(F_{S_{\epsilon },{\hat{S}}_{\epsilon }}\right)$ *uniformly over* k∈N *.*

**Proof.** To begin the proof, for $\left(s^{\left(k\right)},{\hat{s}}^{\left(k\right)}\right)\in R^{2k}$, define

(A22) $$f_{k,n}\left(s^{\left(k\right)},{\hat{s}}^{\left(k\right)}\right)≜\frac{p_{S_{n}^{\left(k\right)}|{\hat{S}}_{n}^{\left(k\right)}}\left(s^{\left(k\right)}|{\hat{s}}^{\left(k\right)}\right)}{p_{S_{n}^{\left(k\right)}}\left(s^{\left(k\right)}\right)},\;f_{k,\epsilon }\left(s^{\left(k\right)},{\hat{s}}^{\left(k\right)}\right)≜\frac{p_{S_{\epsilon }^{\left(k\right)}|{\hat{S}}_{\epsilon }^{\left(k\right)}}\left(s^{\left(k\right)}|{\hat{s}}^{\left(k\right)}\right)}{p_{S_{\epsilon }^{\left(k\right)}}\left(s^{\left(k\right)}\right)}.$$

Now, we recall the backward channel relationship (20):

(A23) $$S_{n}^{\left(k\right)}={\hat{S}}_{n}^{\left(k\right)}+W_{n}^{\left(k\right)},$$

where ${\hat{S}}_{n}^{\left(k\right)}$ and $W_{n}^{\left(k\right)}$ are mutually independent zero-mean, Gaussian random vectors with independent entries, corresponding to the optimal compression process and its respective distortion. From this relationship we obtain

(A24) $$\begin{array}{cc} p_{S_{n}^{\left(k\right)}|{\hat{S}}_{n}^{\left(k\right)}}\left(s^{\left(k\right)}|{\hat{s}}^{\left(k\right)}\right) & \overset{\left(a\right)}{=}p_{{\hat{S}}_{n}^{\left(k\right)}+W_{n}^{\left(k\right)}|{\hat{S}}_{n}^{\left(k\right)}}\left(s^{\left(k\right)}|{\hat{s}}^{\left(k\right)}\right)\\ \; & =p_{W_{n}^{\left(k\right)}|{\hat{S}}_{n}^{\left(k\right)}}\left(s^{\left(k\right)}-{\hat{s}}^{\left(k\right)}|{\hat{s}}^{\left(k\right)}\right)\\ \; & \overset{\left(b\right)}{=}p_{W_{n}^{\left(k\right)}}\left(s^{\left(k\right)}-{\hat{s}}^{\left(k\right)}\right), \end{array}$$

where (a) follows since $S_{n}^{\left(k\right)}={\hat{S}}_{n}^{\left(k\right)}+W_{n}^{\left(k\right)}$, see (A23), and (b) follows since $W_{n}^{\left(k\right)}$ and ${\hat{S}}_{n}^{\left(k\right)}$ are mutually independent. The joint PDF of $S_{n}^{\left(k\right)}$ and ${\hat{S}}_{n}^{\left(k\right)}$ can be expressed via the conditional PDF as:

(A25) $$\begin{array}{c} p_{S_{n}^{\left(k\right)},{\hat{S}}_{n}^{\left(k\right)}}\left(s^{\left(k\right)},{\hat{s}}^{\left(k\right)}\right)=p_{S_{n}^{\left(k\right)}|{\hat{S}}_{n}^{\left(k\right)}}\left(s^{\left(k\right)}|{\hat{s}}^{\left(k\right)}\right)\cdot p_{{\hat{S}}_{n}^{\left(k\right)}}\left({\hat{s}}^{\left(k\right)}\right)\overset{\left(a\right)}{=}p_{W_{n}^{\left(k\right)}}\left(s^{\left(k\right)}-{\hat{s}}^{\left(k\right)}\right)\cdot p_{{\hat{S}}_{n}^{\left(k\right)}}\left({\hat{s}}^{\left(k\right)}\right), \end{array}$$

where (a) follows from (A24). Since ${\hat{S}}_{n}^{\left(k\right)}$ and $W_{n}^{\left(k\right)}$ are Gaussian and mutually independent and since the product of two multivariate Gaussian PDFs is also a multivariate Gaussian PDF ([36] Section 3), it follows from (A25) that $S_{n}^{\left(k\right)}$ and ${\hat{S}}_{n}^{\left(k\right)}$ are jointly Gaussian. Following the mutual independence of $W_{n}^{\left(k\right)}$ and ${\hat{S}}_{n}^{\left(k\right)}$, the right hand side (RHS) of (A25) is also equivalent to the joint PDF of ${\left[{\left(W_{n}^{\left(k\right)}\right)}^{T},{\left({\hat{S}}_{n}^{\left(k\right)}\right)}^{T}\right]}^{T}$ denoted by $p_{W_{n}^{\left(k\right)},{\hat{S}}_{n}^{\left(k\right)}}\left(s^{\left(k\right)}-{\hat{s}}^{\left(k\right)},{\hat{s}}^{\left(k\right)}\right)$. Now, from (A24), the assumption $\lim_{n\to \infty }p_{W_{n}^{\left(k\right)}}\left(w^{\left(k\right)}\right)=p_{W_{\epsilon }^{\left(k\right)}}\left(w^{\left(k\right)}\right)$ implies that a limit exists for the conditional PDF $p_{S_{n}^{\left(k\right)}|{\hat{S}}_{n}^{\left(k\right)}}\left(s^{\left(k\right)}|{\hat{s}}^{\left(k\right)}\right)$, this we denote by $p_{S_{\epsilon }^{\left(k\right)}|{\hat{S}}_{\epsilon }^{\left(k\right)}}\left(s^{\left(k\right)}|{\hat{s}}^{\left(k\right)}\right)$. Combining this with the assumption $\lim_{n\to \infty }p_{{\hat{S}}_{n}^{\left(k\right)}}\left({\hat{s}}^{\left(k\right)}\right)=p_{{\hat{S}}_{\epsilon }^{\left(k\right)}}\left({\hat{s}}^{\left(k\right)}\right)$, we have that,

(A26) $$\begin{array}{cc} \lim_{n\to \infty }p_{S_{n}^{\left(k\right)},{\hat{S}}_{n}^{\left(k\right)}}\left(s^{\left(k\right)},{\hat{s}}^{\left(k\right)}\right) & =\lim_{n\to \infty }\left(p_{S_{n}^{\left(k\right)}|{\hat{S}}_{n}^{\left(k\right)}}\left(s^{\left(k\right)}|{\hat{s}}^{\left(k\right)}\right)\cdot p_{{\hat{S}}_{n}^{\left(k\right)}}\left({\hat{s}}^{\left(k\right)}\right)\right)\\ \; & \overset{\left(a\right)}{=}\lim_{n\to \infty }\left(p_{W_{n}^{\left(k\right)}}\left(s^{\left(k\right)}-{\hat{s}}^{\left(k\right)}\right)\cdot p_{{\hat{S}}_{n}^{\left(k\right)}}\left({\hat{s}}^{\left(k\right)}\right)\right)\\ \; & \overset{\left(b\right)}{=}\lim_{n\to \infty }\left(p_{W_{n}^{\left(k\right)}}\left(s^{\left(k\right)}-{\hat{s}}^{\left(k\right)}\right)\right)\cdot \lim_{n\to \infty }\left(p_{{\hat{S}}_{n}^{\left(k\right)}}\left({\hat{s}}^{\left(k\right)}\right)\right)\\ \; & =p_{S_{\epsilon }^{\left(k\right)}|{\hat{S}}_{\epsilon }^{\left(k\right)}}\left(s^{\left(k\right)}|{\hat{s}}^{\left(k\right)}\right)\cdot p_{{\hat{S}}_{\epsilon }^{\left(k\right)}}\left({\hat{s}}^{\left(k\right)}\right)\\ \; & =p_{S_{\epsilon }^{\left(k\right)},{\hat{S}}_{\epsilon }^{\left(k\right)}}\left(s^{\left(k\right)},{\hat{s}}^{\left(k\right)}\right), \end{array}$$

where (a) follows from (A24), and (b) follows since the limit for each sequence in the product exists ([31] Theorem 3.3); Convergence is uniform in ${\left({\left({\hat{s}}^{\left(k\right)}\right)}^{T},{\left(s^{\left(k\right)}\right)}^{T}\right)}^{T}\in R^{2k}$ and k∈N, as each sequence converges uniformly in k∈N ([31] Page 165). Observe that the joint PDF for the zero-mean Gaussian random vectors $\left[S_{n}^{\left(k\right)},{\hat{S}}_{n}^{\left(k\right)}\right]$ is given by the general expression:

(A27) $$p_{S_{n}^{\left(k\right)},{\hat{S}}_{n}^{\left(k\right)}}\;\left(s^{\left(k\right)},{\hat{s}}^{\left(k\right)}\right)\;=\;{\left(\mathrm{Det}\left(2\pi {\tilde{C}}_{n}^{\left(2k\right)}\right)\right)}^{-\frac{1}{2}}\;\exp\left(-\frac{1}{2}\left[{\left({\hat{s}}^{\left(k\right)}\right)}^{T}\;\;,{\left(s^{\left(k\right)}\right)}^{T}\right]{\left({\tilde{C}}_{n}^{\left(2k\right)}\right)}^{-1}\;{\left[{\left({\hat{s}}^{\left(k\right)}\right)}^{T}\;\;,{\left(s^{\left(k\right)}\right)}^{T}\right]}^{T}\right),$$

where ${\tilde{C}}_{n}^{\left(2k\right)}$ denotes the joint covariance matrix of ${\left[{\left({\hat{S}}_{n}^{\left(k\right)}\right)}^{T},{\left(S_{n}^{\left(k\right)}\right)}^{T}\right]}^{T}$. From (A27) we note that $p_{S_{n}^{\left(k\right)},{\hat{S}}_{n}^{\left(k\right)}}\left(s^{\left(k\right)},{\hat{s}}^{\left(k\right)}\right)$ is a continuous mapping of ${\tilde{C}}_{n}^{\left(2k\right)}$ with respect to the index *n*, see ([17] Lemma B.1). Hence the convergence in (A26) of $p_{S_{n}^{\left(k\right)},{\hat{S}}_{n}^{\left(k\right)}}\left(s^{\left(k\right)},{\hat{s}}^{\left(k\right)}\right)$ as n→∞ directly implies the convergence of ${\tilde{C}}_{n}^{\left(2k\right)}$ as n→∞ to a limit which we denote by ${\tilde{C}}_{\epsilon }^{\left(2k\right)}$. It therefore follows that the limit function $p_{S_{\epsilon }^{\left(k\right)},{\hat{S}}_{\epsilon }^{\left(k\right)}}\left(s^{\left(k\right)},{\hat{s}}^{\left(k\right)}\right)$ corresponds to the PDF of a Gaussian vector with the covariance matrix ${\tilde{C}}_{\epsilon }^{\left(2k\right)}$. The joint PDF for the zero-mean Gaussian random vectors $\left[W_{n}^{\left(k\right)},{\hat{S}}_{n}^{\left(k\right)}\right]$ can be obtained using their mutual independence as:

(A28) $$\begin{array}{c} p_{W_{n}^{\left(k\right)},{\hat{S}}_{n}^{\left(k\right)}}\left(s^{\left(k\right)}-{\hat{s}}^{\left(k\right)},{\hat{s}}^{\left(k\right)}\right)\\ ={\left(\mathrm{Det}\left(2\pi \Sigma_{n}^{\left(2k\right)}\right)\right)}^{\frac{1}{2}}\;\exp\left(-\frac{1}{2}\left[{\left(s^{\left(k\right)}\;-\;{\hat{s}}^{\left(k\right)}\right)}^{T},{\left({\hat{s}}^{\left(k\right)}\right)}^{T}\right]{\left(\Sigma_{n}^{\left(2k\right)}\right)}^{-\;1}\;{\left[{\left(s^{\left(k\right)}\;-\;{\hat{s}}^{\left(k\right)}\right)}^{T},{\left({\hat{s}}^{\left(k\right)}\right)}^{T}\right]}^{T}\right), \end{array}$$

where $\Sigma_{n}^{\left(2k\right)}$ denotes the joint covariance matrix of ${\left[{\left(W_{n}^{\left(k\right)}\right)}^{T},{\left({\hat{S}}_{n}^{\left(k\right)}\right)}^{T}\right]}^{T}$. Since the vectors $W_{n}^{\left(k\right)}$ and ${\hat{S}}_{n}^{\left(k\right)}$ are zero-mean, mutually independent and, by the relationship (20), each vector has independent entries, it follows that $\Sigma_{n}^{\left(2k\right)}$ is a diagonal matrix with each diagonal element taking the value of the corresponding temporal variance at the respective index i∈{1,2,…,k}. i.e.,

(A29) $$\begin{array}{cc} \Sigma_{n}^{\left(2k\right)} & ≜E\left\{{\left({\left(W_{n}^{\left(k\right)}\right)}^{T},{\left({\hat{S}}_{n}^{\left(k\right)}\right)}^{T}\right)}^{T}\cdot \left({\left(W_{n}^{\left(k\right)}\right)}^{T},{\left({\hat{S}}_{n}^{\left(k\right)}\right)}^{T}\right)\right\}\\ \; & =\mathrm{diag}\left(E\left\{{\left(W_{n}\left[1\right]\right)}^{2}\right\},E\left\{{\left(W_{n}\left[2\right]\right)}^{2}\right\},\ldots ,E\left\{{\left(W_{n}\left[k\right]\right)}^{2}\right\},\sigma_{{\hat{S}}_{n}}^{2}\left[1\right],\sigma_{{\hat{S}}_{n}}^{2}\left[2\right]\ldots ,\sigma_{{\hat{S}}_{n}}^{2}\left[k\right]\right). \end{array}$$

The convergence of $p_{W_{n}^{\left(k\right)},{\hat{S}}_{n}^{\left(k\right)}}\left(s^{\left(k\right)}-{\hat{s}}^{\left(k\right)},{\hat{s}}^{\left(k\right)}\right)$, from (A26), implies a convergence of the diagonal elements in (A29) as n→∞. Hence $\Sigma_{n}^{\left(2k\right)}$ converges as n→∞ to a diagonal joint covariance matrix which we denote by $\Sigma_{\epsilon }^{\left(2k\right)}$. This further implies that the limiting vectors $W_{\epsilon }^{\left(k\right)}$ and ${\hat{S}}_{\epsilon }^{\left(k\right)}$ are zero-mean, mutually independent and each vector has independent entries in i∈[1,2,…,k]. Relationship (A26) implies that the joint limit distribution satisfies $p_{S_{\epsilon }^{\left(k\right)},{\hat{S}}_{\epsilon }^{\left(k\right)}}\left(s^{\left(k\right)},{\hat{s}}^{\left(k\right)}\right)=p_{{\hat{S}}_{\epsilon }^{\left(k\right)}}\left({\hat{s}}^{\left(k\right)}\right)p_{W_{\epsilon }^{\left(k\right)}}\left(s^{\left(k\right)}-{\hat{s}}^{\left(k\right)}\right)$. Consequently, we can define an asymptotic backward channel that satisfies (A26) via the expression:

(A30) $$S_{\epsilon }^{\left(k\right)}\left[i\right]={\hat{S}}_{\epsilon }^{\left(k\right)}\left[i\right]+W_{\epsilon }^{\left(k\right)}\left[i\right].$$

Next, by convergence of the joint PDF $p_{W_{n}^{\left(k\right)}}\left(s^{\left(k\right)}-{\hat{s}}^{\left(k\right)}\right)\cdot p_{{\hat{S}}_{n}^{\left(k\right)}}\left({\hat{s}}^{\left(k\right)}\right)$ uniformly in k∈N and in ${\left({\left(s^{\left(k\right)}\right)}^{T},{\left({\hat{s}}^{\left(k\right)}\right)}^{T}\right)}^{T}\in R^{2k}$, it follows from ([35] Theorem 1) that ${\left[{\left({\hat{S}}_{n}^{\left(k\right)}\right)}^{T},{\left(W_{n}^{\left(k\right)}\right)}^{T}\right]}^{T}\overset{\left(dist.\right)}{\underset{n\to \infty }{⟶}}{\left[{\left({\hat{S}}_{\epsilon }^{\left(k\right)}\right)}^{T},{\left(W_{\epsilon }^{\left(k\right)}\right)}^{T}\right]}^{T}$ and the convergence is uniform in k∈N and in ${\left({\left(s^{\left(k\right)}\right)}^{T},{\left({\hat{s}}^{\left(k\right)}\right)}^{T}\right)}^{T}\in R^{2k}$. Then, by the continuous mapping theorem (CMT) ([37] Theorem 7.7), we have

$$\begin{array}{c} {\left[{\left(S_{n}^{\left(k\right)}\right)}^{T},{\left({\hat{S}}_{n}^{\left(k\right)}\right)}^{T}\right]}^{T}\;=\;{\left[{\left({\hat{S}}_{n}^{\left(k\right)}\;+\;W_{n}^{\left(k\right)}\right)}^{T},{\left({\hat{S}}_{n}^{\left(k\right)}\right)}^{T}\right]}^{T}\overset{\left(dist.\right)}{\underset{n\to \infty }{⟶}}{\left[{\left({\hat{S}}_{\epsilon }^{\left(k\right)}\;+\;W_{\epsilon }^{\left(k\right)}\right)}^{T},{\left({\hat{S}}_{\epsilon }^{\left(k\right)}\right)}^{T}\right]}^{T}\;=\;{\left[{\left(S_{\epsilon }^{\left(k\right)}\right)}^{T},{\left({\hat{S}}_{\epsilon }^{\left(k\right)}\right)}^{T}\right]}^{T}. \end{array}$$

Now, using the extended CMT ([37] Theorem 7.24), we will show that $f_{k,n}\left(S_{n}^{\left(k\right)},{\hat{S}}_{n}^{\left(k\right)}\right)\overset{\left(dist.\right)}{\underset{n\to \infty }{⟶}}f_{k,\epsilon }\left(S_{\epsilon }^{\left(k\right)},{\hat{S}}_{\epsilon }^{\left(k\right)}\right)$ for each k∈N, following the same approach as in the proof of ([17] Lemma B.2). Then, since ${\tilde{Z}}_{k,n}^{\prime }\left(F_{S_{n},{\hat{S}}_{n}}^{\mathrm{opt}}\right)=\frac{1}{k}\log f_{k,n}\left(S_{n}^{\left(k\right)},{\hat{S}}_{n}^{\left(k\right)}\right)$ and $Z_{k}^{\prime }\left(F_{S_{\epsilon },{\hat{S}}_{\epsilon }}\right)=\frac{1}{k}\log f_{k,\epsilon }\left(S_{\epsilon }^{\left(k\right)},{\hat{S}}_{\epsilon }^{\left(k\right)}\right)$, we conclude that ${\tilde{Z}}_{k,n}^{\prime }\left(F_{S_{n},{\hat{S}}_{n}}^{\mathrm{opt}}\right)\overset{\left(dist.\right)}{\underset{n\to \infty }{⟶}}Z_{k}^{\prime }\left(F_{S_{\epsilon },{\hat{S}}_{\epsilon }}\right)$, where it also follows from the proof of ([17] Lemma B.2) that the convergence is uniform in k∈N. Specifically, to prove that $f_{k,n}\left(S_{n}^{\left(k\right)},{\hat{S}}_{n}^{\left(k\right)}\right)\overset{\left(dist.\right)}{\underset{n\to \infty }{⟶}}f_{k,\epsilon }\left(S_{\epsilon }^{\left(k\right)},{\hat{S}}_{\epsilon }^{\left(k\right)}\right)$, we will show that the following two properties hold:

P1 The distribution of ${\left[{\left(S_{\epsilon }^{\left(k\right)}\right)}^{T},{\left({\hat{S}}_{\epsilon }^{\left(k\right)}\right)}^{T}\right]}^{T}$ is separable (as defined in ([37] Pg. 101)).
P2 For any convergent sequence ${\left({\left(s_{n}^{\left(k\right)}\right)}^{T},{\left({\hat{s}}_{n}^{\left(k\right)}\right)}^{T}\right)}^{T}\in R^{2k}$ such that $\lim_{n\to \infty }\left(s_{n}^{\left(k\right)},{\hat{s}}_{n}^{\left(k\right)}\right)=\left(s_{\epsilon }^{\left(k\right)},{\hat{s}}_{\epsilon }^{\left(k\right)}\right)$, then $\lim_{n\to \infty }f_{k,n}\left(s_{n}^{\left(k\right)},{\hat{s}}_{n}^{\left(k\right)}\right)=f_{k,\epsilon }\left(s_{\epsilon }^{\left(k\right)},{\hat{s}}_{\epsilon }^{\left(k\right)}\right)$.

To prove property P1, we show that $U^{\left(k\right)}≜{\left[{\left(S_{\epsilon }^{\left(k\right)}\right)}^{T},{\left({\hat{S}}_{\epsilon }^{\left(k\right)}\right)}^{T}\right]}^{T}$ (here we misuse the dimension notation as $U^{\left(k\right)}$ denotes a 2k-dimensional vector) is separable ([37] Pg. 101), i.e., we show that ∀η>0, there exists β>0 such that $\mathrm{Pr}\left(∥U^{\left(k\right)}{∥}^{2}>\beta \right)<\eta$. To that aim, recall first that by Markov’s inequality ([29] Pg. 114), it follows that $\mathrm{Pr}U^{\left(k\right)}∥{}^{2}>\beta )<\frac{1}{\beta }E\left\{{∥U^{\left(k\right)}∥}^{2}\right\}$. For the asynchronously sampled source process, we note that $\sigma_{S_{\epsilon }}^{2}\left[i\right]≜E\left\{{\left(S_{\epsilon }\left[i\right]\right)}^{2}\right\}\in \left[0,\max_{0\leq t\leq T_{\mathrm{ps}}}\sigma_{S_{c}}^{2}\left(t\right)\right]$. By the independence of $W_{\epsilon }^{\left(k\right)}$ and ${\hat{S}}_{\epsilon }^{\left(k\right)}$, and by the fact that their mean is zero, we have, from (A30) that $E\left\{{\left(S_{\epsilon }\left[i\right]\right)}^{2}\right\}=E\left\{{\left({\hat{S}}_{\epsilon }\left[i\right]\right)}^{2}\right\}+E\left\{{\left(W_{\epsilon }\left[i\right]\right)}^{2}\right\}\leq \max_{0\leq t\leq T_{\mathrm{ps}}}\sigma_{S_{c}}^{2}\left(t\right)$; Hence $E\left\{{\left({\hat{S}}_{\epsilon }\left[i\right]\right)}^{2}\right\}\leq \max_{0\leq t\leq T_{\mathrm{ps}}}\sigma_{S_{c}}^{2}\left(t\right)$, and $E\left\{{\left(W_{\epsilon }\left[i\right]\right)}^{2}\right\}\leq \max_{0\leq t\leq T_{\mathrm{ps}}}\sigma_{S_{c}}^{2}\left(t\right)$. This further implies that $E\left\{{∥U^{\left(k\right)}∥}^{2}\right\}=E\left\{{∥{\left[{\left(S_{\epsilon }^{\left(k\right)}\right)}^{T},{\left({\hat{S}}_{\epsilon }^{\left(k\right)}\right)}^{T}\right]}^{T}∥}^{2}\right\}\leq 2\cdot k\cdot \max_{0\leq t\leq T_{\mathrm{ps}}}\sigma_{S_{c}}^{2}\left(t\right)$; therefore for each $\beta >\frac{1}{\eta }E\left\{{∥U^{\left(k\right)}∥}^{2}\right\}$ we have that $\mathrm{Pr}\left({∥U^{\left(k\right)}∥}^{2}>\beta \right)<\eta$, and thus $U^{\left(k\right)}$ is separable. By the assumption in this lemma it follows that ∀η>0 there exists $n_{0}\left(\eta \right)>0$ such that for all $n>n_{0}\left(\eta \right)$ we have that $\forall w^{\left(k\right)}\in R^{k}$, $|p_{W_{n}^{\left(k\right)}}\left(w^{\left(k\right)}\right)-p_{W_{\epsilon }^{\left(k\right)}}\left(w^{\left(k\right)}\right)|<\eta$, for all sufficiently large k∈N. Consequently, for all ${\left({\left(s^{\left(k\right)}\right)}^{T},{\left({\hat{s}}^{\left(k\right)}\right)}^{T}\right)}^{T}\in R^{2k}$, $n>n_{0}\left(\eta \right)$ and a sufficiently large k∈N, it follows from (A24) that

(A31) $$\begin{array}{cc} \;\left|p_{S_{n}^{\left(k\right)}|{\hat{S}}_{n}^{\left(k\right)}}\left(s^{\left(k\right)}|{\hat{s}}^{\left(k\right)}\right)-p_{S_{\epsilon }^{\left(k\right)}|{\hat{S}}_{\epsilon }^{\left(k\right)}}\left(s^{\left(k\right)}|{\hat{s}}^{\left(k\right)}\right)\right| & =\left|p_{W_{n}^{\left(k\right)}}\left(s^{\left(k\right)}-{\hat{s}}^{\left(k\right)}\right)-p_{W_{\epsilon }^{\left(k\right)}}\left(s^{\left(k\right)}-{\hat{s}}^{\left(k\right)}\right)\right|<\eta . \end{array}$$

Following the continuity of $p_{S_{n}^{\left(k\right)}|{\hat{S}}_{n}^{\left(k\right)}}\left(s^{\left(k\right)}|{\hat{s}}^{\left(k\right)}\right)$ and of $p_{S_{n}^{\left(k\right)}}\left(s^{\left(k\right)}\right)$, $f_{k,n}\left(s^{\left(k\right)},{\hat{s}}^{\left(k\right)}\right)$ is also continuous ([31] Theorem 4.9); hence, when $\lim_{n\to \infty }\left(s_{n}^{\left(k\right)},{\hat{s}}_{n}^{\left(k\right)}\right)=\left(s^{\left(k\right)},{\hat{s}}^{\left(k\right)}\right)$, then $\lim_{n\to \infty }f_{k,n}\left(s_{n}^{\left(k\right)},{\hat{s}}_{n}^{\left(k\right)}\right)=f_{k,\epsilon }\left(s^{\left(k\right)},{\hat{s}}^{\left(k\right)}\right)$. This satisfies condition P2 for the extended CMT; Therefore, by the extended CMT, we have that $f_{k,n}\left(S_{n}^{\left(k\right)},{\hat{S}}_{n}^{\left(k\right)}\right)\overset{\left(dist.\right)}{\underset{n\to \infty }{⟶}}f_{k,\epsilon }\left(S_{\epsilon }^{\left(k\right)},{\hat{S}}_{\epsilon }^{\left(k\right)}\right)$. Since the RVs ${\tilde{Z}}_{k,n}^{\prime }\left(F_{S_{n},{\hat{S}}_{n}}^{\mathrm{opt}}\right)$ and $Z_{k,\epsilon }^{\prime }\left(F_{S_{\epsilon },{\hat{S}}_{\epsilon }}\right)$, defined in (A17), are also continuous mappings of $f_{k,n}\left(S_{n}^{\left(k\right)},{\hat{S}}_{n}^{\left(k\right)}\right)$ and of $f_{k,\epsilon }\left(S_{\epsilon }^{\left(k\right)},{\hat{S}}_{\epsilon }^{\left(k\right)}\right)$, respectively, it follows from the CMT ([37] Theorem 7.7) that ${\tilde{Z}}_{k,n}^{\prime }\left(F_{S_{n},{\hat{S}}_{n}}^{\mathrm{opt}}\right)\overset{\left(dist.\right)}{\underset{n\to \infty }{⟶}}Z_{k,\epsilon }^{\prime }\left(F_{S_{\epsilon },{\hat{S}}_{\epsilon }}\right)$. Finally, to prove that the convergence ${\tilde{Z}}_{k,n}^{\prime }\left(F_{S_{n},{\hat{S}}_{n}}^{\mathrm{opt}}\right)\overset{\left(dist.\right)}{\underset{n\to \infty }{⟶}}Z_{k,\epsilon }^{\prime }\left(F_{S_{\epsilon },{\hat{S}}_{\epsilon }}\right)$ is uniform in k∈N, we note that as ${\hat{S}}_{n}^{\left(k\right)}$ and ${\hat{S}}_{\epsilon }^{\left(k\right)}$ have independent entries, and the backward channels (21) and (A30) are memoryless. Hence, it follows from the proof of ([17] Lemma B.2), that the characteristic function of the RV $k\cdot {\tilde{Z}}_{k,n}^{\prime }\left(F_{S_{n},{\hat{S}}_{n}}^{\mathrm{opt}}\right)$ which is denoted by $\Phi_{k\cdot {\tilde{Z}}_{k,n}}\left(\alpha \right)≜E\left\{e^{j\cdot \alpha \cdot k\cdot {\tilde{Z}}_{k,n}}\right\}$ converges to the characteristic function of $k\cdot Z_{k,\epsilon }^{\prime }\left(F_{S_{\epsilon },{\hat{S}}_{\epsilon }}\right)$, denoted by $\Phi_{k\cdot Z_{k,\epsilon }}\left(\alpha \right)$, uniformly over k∈N. Thus, for all sufficiently small η>0, $\exists k_{0}\in N,n_{0}\left(\eta ,k_{0}\right)\in N$ such that $\forall n>n_{0}\left(\eta ,k_{0}\right)$, and $\forall k>k_{0}$

(A32) $$|\Phi_{k\cdot {\tilde{Z}}_{k,n}}\left(\alpha \right)-\Phi_{k\cdot Z_{k,\epsilon }}\left(\alpha \right)|<\eta ,\;\forall \alpha \cdot \in R.$$

Hence, following Lévy’s convergence theorem ([38] Theorem 18.1) we conclude that $k\cdot {\tilde{Z}}_{k,n}^{\prime }\left(F_{S_{n},{\hat{S}}_{n}}^{\mathrm{opt}}\right)\overset{\left(dist.\right)}{\underset{n\to \infty }{⟶}}k\cdot Z_{k,\epsilon }^{\prime }\left(F_{S_{\epsilon },{\hat{S}}_{\epsilon }}\right)$ and that this convergence is uniform for sufficiently large *k*. Finally, since the CDFs of $k\cdot {\tilde{Z}}_{k,n}^{\prime }\left(F_{S_{n},{\hat{S}}_{n}}^{\mathrm{opt}}\right)$ and $k\cdot Z_{k,\epsilon }^{\prime }\left(F_{S_{\epsilon },{\hat{S}}_{\epsilon }}\right)$ obtained at α∈R are equivalent to the CDFs of ${\tilde{Z}}_{k,n}^{\prime }\left(F_{S_{n},{\hat{S}}_{n}}^{\mathrm{opt}}\right)$ and $Z_{k,\epsilon }^{\prime }\left(F_{S_{\epsilon },{\hat{S}}_{\epsilon }}\right)$ obtained at $\frac{\alpha }{k}\in R$ respectively, we can conclude that ${\tilde{Z}}_{k,n}^{\prime }\left(F_{S_{n},{\hat{S}}_{n}}^{\mathrm{opt}}\right)\overset{\left(dist.\right)}{\underset{n\to \infty }{⟶}}Z_{k,\epsilon }^{\prime }\left(F_{S_{\epsilon },{\hat{S}}_{\epsilon }}\right)$, uniformly in k∈N. □

The following convergence lemma A5 corresponds to ([17] Lemma B.3),

**Lemma** **A5.**
*Let* n∈N *be given. Every subsequence of* ${\left\{{\tilde{Z}}_{k,n}^{\prime }\left(F_{{\hat{S}}_{n},S_{n}}^{\mathrm{opt}}\right)\right\}}_{k\in N}$ *, indexed by* $k_{l}$ *, converges in distribution, in the limit as* l→∞ *, to a finite deterministic scalar.*

**Proof.** Recall that the RVs ${\tilde{Z}}_{k,n}^{\prime }\left(F_{{\hat{S}}_{n},S_{n}}^{\mathrm{opt}}\right)$ represent the mutual information density rate between *k* samples of the source process $S_{n}\left[i\right]$ and the corresponding samples of its reproduction process ${\hat{S}}_{n}\left[i\right]$, where these processes are jointly distributed via the Gaussian distribution measure $F_{{\hat{S}}_{n},S_{n}}^{\mathrm{opt}}$. Further, recall that the relationship between the source signal and the reproduction process which achieves the RDF can be described via the backward channel in (21) for a Gaussian source. The channel (21) is a memoryless additive WSCS Gaussian noise channel with period $p_{n}$, thus, by [21], it can be equivalently represented as a $p_{n}\times 1$ multivariate memoryless additive stationary Gaussian noise channel, which is an information stable channel ([39] Section 1.5). For such channels in which the source and its reproduction obey the RDF-achieving joint distribution $F_{S_{n},{\hat{S}}_{n}}^{\mathrm{opt}}$, the mutual information density rate converges as *k* increases, almost surely, to the finite and deterministic mutual information rate ([14] Theorem 5.9.1). Since almost sure convergence implies convergence in distribution ([37] Lemma 7.21), this proves the lemma. □

### Appendix D.3. Showing that $R_{\epsilon }\left(D\right)=\underset{n\to \infty }{\lim\sup}R_{n}\left(D\right)$

This section completes the proof to Theorem 4. We note from (14) that the RDF for the source process $S_{n}\left[i\right]$ (for fixed length coding and MSE distortion measure) is given by:

(A33) $$R_{n}\left(D\right)=\underset{F_{{\hat{S}}_{n},S_{n}}:{\bar{d}}_{S}\left(F_{{\hat{S}}_{n},S_{n}}\right)\leq D}{\inf}\left\{p-\underset{k\to \infty }{\lim\sup}{\tilde{Z}}_{k,n}^{\prime }\left(F_{{\hat{S}}_{n},S_{n}}^{\mathrm{opt}}\right)\right\},$$

where ${\bar{d}}_{S}\left(F_{{\hat{S}}_{n},S_{n}}\right)=\underset{k\to \infty }{\lim\sup}\frac{1}{k}E\left\{∥S_{n}^{\left(k\right)}-{\hat{S}}_{n}^{\left(k\right)}{∥}^{2}\right\}$.

We now state the following lemma characterizing the asymptotic statistics of the optimal reconstruction ${\hat{S}}_{n}^{\left(k\right)}$ process and the respective noise process $W_{n}^{\left(k\right)}$ used in the backward channel relationship (21):

**Lemma** **A6.**
*Consider the RDF-achieving distribution with distortion D for compression of a vector Gaussian source process* $S_{n}^{\left(k\right)}$ *characterized by the backward channel *(21)*. Then, there exists a subsequence in the index* n∈N *denoted* $n_{1}<n_{2}<\ldots$ *, such that for the RDF-achieving distribution, the sequences of reproduction vectors* ${\left\{{\hat{S}}_{n_{l}}^{\left(k\right)}\right\}}_{l\in N}$ *and backward channel noise vectors* ${\left\{W_{n_{l}}^{\left(k\right)}\right\}}_{l\in N}$ *satisfy that* $\lim_{l\to \infty }p_{{\hat{S}}_{n_{l}}^{\left(k\right)}}\left({\hat{s}}^{\left(k\right)}\right)=p_{{\hat{S}}_{\epsilon }^{\left(k\right)}}\left({\hat{s}}^{\left(k\right)}\right)$ *uniformly in* ${\hat{s}}^{\left(k\right)}\in R^{k}$ *and uniformly with respect to* k∈N *, as well as* $\lim_{l\to \infty }p_{W_{n_{l}}^{\left(k\right)}}\left(w^{\left(k\right)}\right)=p_{W_{\epsilon }^{\left(k\right)}}\left(w^{\left(k\right)}\right)$ *uniformly in* $w^{\left(k\right)}\in R^{k}$ *and uniformly with respect to* k∈N *, where* $p_{{\hat{S}}_{\epsilon }^{\left(k\right)}}\left({\hat{s}}^{\left(k\right)}\right)$ *and* $p_{W_{\epsilon }^{\left(k\right)}}\left(w^{\left(k\right)}\right)$ *are Gaussian PDFs.*

**Proof.** Recall from the analysis of the RDF for WSCS processes that for each n∈N, the marginal distributions of the RDF-achieving reproduction process ${\hat{S}}_{n}\left[i\right]$ and the backward channel noise $W_{n}\left[i\right]$ is Gaussian, memoryless, zero-mean, and with variances $\sigma_{{\hat{S}}_{n}}^{2}\left[i\right]≜E\left\{{\left({\hat{S}}_{n}\left[i\right]\right)}^{2}\right\}$ and

(A34) $$E\left\{{\left(W_{n}\left[i\right]\right)}^{2}\right\}=\sigma_{S_{n}}^{2}\left[i\right]-\sigma_{{\hat{S}}_{n}}^{2}\left[i\right],$$

respectively. Consequently, the sequences of reproduction vectors ${\left\{{\hat{S}}_{n}^{\left(k\right)}\right\}}_{n\in N}$ and backward channel noise vectors ${\left\{W_{n}^{\left(k\right)}\right\}}_{n\in N}$ are zero-mean Gaussian with independent entries for each k∈N. Since $\sigma_{S_{n}}^{2}\left[i\right]\leq \max_{t\in R}\sigma_{S_{c}}^{2}\left(t\right)$, then, from (A34), it follows that $\sigma_{{\hat{S}}_{n}}^{2}\left[i\right]$ is also bounded in the interval $\left[0,\max_{t\in R}\sigma_{S_{c}}^{2}\left(t\right)\right]$ for all n∈N. Therefore, by Bolzano-Weierstrass theorem ([31] Theorem 2.42), $\sigma_{{\hat{S}}_{n}}^{2}\left[i\right]$ has a convergent subsequence, and we let $n_{1}<n_{2}<\ldots$ denote the indexes of this convergent subsequence and let the limit of the subsequence be denoted by $\sigma_{{\hat{S}}_{\epsilon }}^{2}\left[i\right]$. From the CMT, as applied in the proof of ([17] Lemma B.1), the convergence $\sigma_{{\hat{S}}_{n_{l}}}^{2}\left[i\right]\underset{l\to \infty }{⟶}\sigma_{{\hat{S}}_{\epsilon }}^{2}\left[i\right]$ for each i∈N implies that the subsequence of PDFs $p_{{\hat{S}}_{n_{l}}^{\left(k\right)}}\left({\hat{s}}^{\left(k\right)}\right)$ corresponding to the memoryless Gaussian random vectors ${\left\{{\hat{S}}_{n_{l}}^{\left(k\right)}\right\}}_{l\in N}$ converges as l→∞ to a Gaussian PDF which we denote by $p_{{\hat{S}}_{\epsilon }^{\left(k\right)}}\left({\hat{s}}^{\left(k\right)}\right)$, and the convergence of $p_{{\hat{S}}_{n_{l}}^{\left(k\right)}}\left({\hat{s}}^{\left(k\right)}\right)$ is uniform in $s^{\left(k\right)}$ for any fixed k∈N. By Remark 2, it holds that $W_{n}\left[i\right]$ is a memoryless stationary process with variance $E\left\{{\left(W_{n}\left[i\right]\right)}^{2}\right\}=D$ and by Equation (A34), $\sigma_{{\hat{S}}_{n}}^{2}\left[i\right]=\sigma_{S_{n}}^{2}\left[i\right]-D$. Hence by Assumption A1 and by the proof of ([17] Lemma B.1), it follows that for a fixed η>0 and $k_{0}\in N$, $\exists n_{0}\left(\eta ,k_{0}\right)$ such that for all $n>n_{0}\left(\eta ,k_{0}\right)$ and for all sufficiently large *k*, it holds that $|p_{{\hat{S}}_{n_{l}}^{\left(k\right)}}\left({\hat{s}}^{\left(k\right)}\right)-p_{{\hat{S}}_{\epsilon }^{\left(k\right)}}\left({\hat{s}}^{\left(k\right)}\right)|<\eta$ for every ${\hat{s}}^{\left(k\right)}\in R^{k}$. Since $n_{0}\left(\eta ,k_{0}\right)$ does not depend on *k* (only on the fixed $k_{0}$), this implies that the convergence is uniform with respect to k∈N. The fact that $W_{n}\left[i\right]$ is a zero-mean stationary Gaussian process with variance *D* for each n∈N, implies that the sequence of PDFs $p_{W_{n}^{\left(k\right)}}\left(w^{\left(k\right)}\right)$ converges as n→∞ to a Gaussian PDF which we denote by $p_{W^{\left(k\right)}}\left(w^{\left(k\right)}\right)$, hence its subsequence with indices $n_{1}<n_{2}<\ldots$ also converges to $p_{W^{\left(k\right)}}\left(w^{\left(k\right)}\right)$. Since $D>\frac{1}{2\pi }$ by Assumption A1 combined with the proof of ([17] Lemma B.1) it follows that this convergence is uniform in $w^{\left(k\right)}$ and in k∈N to $p_{W_{\epsilon }^{\left(k\right)}}\left(w^{\left(k\right)}\right)$. Following the proof of Corollary A1, it holds that the subsequences of the memoryless Gaussian random vectors $\left\{{\hat{S}}_{n_{l}}^{\left(k\right)}\right\}$ and $\left\{W_{n_{l}}^{\left(k\right)}\right\}$ converge in distribution as l→∞ to a Gaussian distribution, and the convergence is uniform in k∈N for any fixed k∈N. Hence, as shown in Lemma A4 the joint distribution ${\left[{\left(S_{n_{l}}^{\left(k\right)}\right)}^{T},{\left({\hat{S}}_{n_{l}}^{\left(k\right)}\right)}^{T}\right]}^{T}\overset{\left(dist.\right)}{\underset{n\to \infty }{⟶}}{\left[{\left(S_{\epsilon }^{\left(k\right)}\right)}^{T},{\left({\hat{S}}_{\epsilon }^{\left(k\right)}\right)}^{T}\right]}^{T}$, and the limit distribution is jointly Gaussian. □

**Lemma** **A7.**
*The RDF of* $\left\{S_{\epsilon }\left[i\right]\right\}$ *satisfies* $R_{\epsilon }\left(D\right)\leq \underset{n\to \infty }{\lim\sup}R_{n}\left(D\right)$ *, and the rate* $\underset{n\to \infty }{\lim\sup}R_{n}\left(D\right)$ *is achievable for the source* $\left\{S_{\epsilon }\left[i\right]\right\}$ *with distortion D when the reproduction process which obeys a Gaussian distribution.*

**Proof.** According to Lemma A6, we note that the sequence of joint distributions ${\left\{F_{S_{n},{\hat{S}}_{n}}^{\mathrm{opt}}\right\}}_{n\in N}$ has a convergent subsequence, i.e., there exists a set of indexes $n_{1}<n_{2}<\ldots$ such that the sequence of distributions with independent entries ${\left\{F_{S_{n_{l}},{\hat{S}}_{n_{l}}}^{\mathrm{opt}}\right\}}_{l\in N}$ converges in the limit l→∞ to a joint Gaussian distribution $F_{S_{\epsilon },{\hat{S}}_{\epsilon }}^{\prime }$ and the convergence is uniform in k∈N. Hence, this satisfies the condition of Lemma A4; This implies that ${\tilde{Z}}_{k,n_{l}}^{\prime }\left(F_{S_{n_{l}},{\hat{S}}_{n_{l}}}^{\mathrm{opt}}\right)\overset{\left(dist.\right)}{\underset{l\to \infty }{⟶}}Z_{k}^{\prime }\left(F_{S_{\epsilon },{\hat{S}}_{\epsilon }}^{\prime }\right)$ uniformly in k∈N. Moreover, by Lemma A5 every subsequence of ${\left\{{\tilde{Z}}_{k,n_{l}}^{\prime }\left(F_{S_{n_{l}},{\hat{S}}_{n_{l}}}^{\mathrm{opt}}\right)\right\}}_{l\in N}$ converges in distribution to a finite deterministic scalar as k→∞. Therefore, by Theorem 3 it holds that

(A35) $$\begin{array}{cc} \lim_{l\to \infty }\left(p-\underset{k\to \infty }{\lim\sup}{\tilde{Z}}_{k,n_{l}}^{\prime }\left(F_{S_{n_{l}},{\hat{S}}_{n_{l}}}^{\mathrm{opt}}\right)\right) & =p-\underset{k\to \infty }{\lim\sup}Z_{k,\epsilon }^{\prime }\left(F_{S_{\epsilon },{\hat{S}}_{\epsilon }}^{\prime }\right)\\ \; & \geq \underset{F_{S_{\epsilon },{\hat{S}}_{\epsilon }}}{\inf}\left\{p-\underset{k\to \infty }{\lim\sup}Z_{k,\epsilon }^{\prime }\left(F_{S_{\epsilon },{\hat{S}}_{\epsilon }}\right)\right\}=R_{\epsilon }\left(D\right). \end{array}$$

From (14) we have that $R_{n}\left(D\right)=p-\underset{k\to \infty }{\lim\sup}{\tilde{Z}}_{k,n}^{\prime }\left(F_{S_{n},{\hat{S}}_{n}}^{\mathrm{opt}}\right)$, then from (A35), it follows that

(A36) $$R_{\epsilon }\left(D\right)\leq \lim_{l\to \infty }R_{n_{l}}\left(D\right)\overset{\left(a\right)}{\leq }\underset{n\to \infty }{\lim\sup}R_{n}\left(D\right),$$

where (a) follows since, by ([31] Definition 3.16), the limit of every subsequence is not greater than the limit superior. Noting that $F_{S_{\epsilon },{\hat{S}}_{\epsilon }}^{\prime }$ is Gaussian by Lemma A6 concludes the proof. □

**Lemma** **A8.**
*The RDF of* $\left\{S_{\epsilon }\left[i\right]\right\}$ *satisfies* $R_{\epsilon }\left(D\right)\geq \underset{n\to \infty }{\lim\sup}R_{n}\left(D\right)$ *.*

**Proof.** To prove this lemma, we first show that for a joint distribution $F_{S_{\epsilon },{\hat{S}}_{\epsilon }}$ which achieves a rate-distortion pair $\left(R_{\epsilon },D\right)$ it holds that $R_{\epsilon }\geq E\left\{Z_{k,\epsilon }^{\prime }\left(F_{S_{\epsilon },{\hat{S}}_{\epsilon }}^{\prime }\right)\right\}$: Recall that $\left(R_{\epsilon },D\right)$ is an achievable rate-distortion pair for the source $\left\{S_{\epsilon }\left[i\right]\right\}$, namely, there exists a sequence of codes $\left\{C_{l}\right\}$ whose rate-distortion approach $\left(R_{\epsilon },D\right)$ when applied to $\left\{S_{\epsilon }\left[i\right]\right\}$, This implies that for any η>0 there exists $l_{0}\left(\eta \right)$ such that $\forall l>l_{0}\left(\eta \right)$ it holds that $C_{l}$ has a code rate $R_{l}=\frac{1}{l}\log_{2}M_{l}$ satisfying $R_{l}\leq R_{\epsilon }+\eta$ by (3). Recalling Definition 5, the source code maps $S_{\epsilon }^{\left(l\right)}$ into a discrete index $J_{l}\in \left\{1,2,\ldots ,M_{l}\right\}$, which is in turn mapped into ${\hat{S}}_{\epsilon }^{\left(l\right)}$, i.e., $S_{\epsilon }^{\left(l\right)}\mapsto J_{l}\mapsto {\hat{S}}_{\epsilon }^{\left(l\right)}$ form a Markov chain. Since $J_{l}$ is a discrete random variable taking values in $\left\{1,2,\ldots ,M_{l}\right\}$, it holds that

(A37) $$\begin{array}{cc} \log_{2}M_{l} & \geq H(J_{l})\\ \; & \overset{\left(a\right)}{\geq }I\left(S_{\epsilon }^{\left(l\right)};J_{l}\right)\\ \; & \overset{\left(b\right)}{\geq }I\left(S_{\epsilon }^{\left(l\right)};{\hat{S}}_{\epsilon }^{\left(l\right)}\right), \end{array}$$

where (a) follows since $I\left(S_{\epsilon }^{\left(l\right)};J_{l}\right)=H\left(J_{l}\right)-H\left(J_{l}|S_{\epsilon }^{\left(l\right)}\right)$ which is not larger than $H(J_{l})$ as $J_{l}$ takes discrete values; while (b) follows from the data processing inequality ([5] Chapter 2.8). Now, (A37) implies that for each $l>l_{0}\left(\eta \right)$, the reproduction obtained using the code $C_{l}$ satisfies $\frac{1}{l}I\left(S_{\epsilon }^{\left(l\right)};{\hat{S}}_{\epsilon }^{\left(l\right)}\right)\leq \frac{1}{l}\log M_{l}\leq R_{\epsilon }+\eta$. Since for every arbitrarily small η→0, this inequality holds for all $l>l_{0}\left(\eta \right)$, i.e., for all sufficiently large *l*, it follows that $R_{\epsilon }\geq \underset{k\to \infty }{\lim\sup}\frac{1}{l}I\left(S_{\epsilon }^{\left(l\right)};{\hat{S}}_{\epsilon }^{\left(l\right)}\right)$. Hence, replacing the blocklength symbol from *l* to *k*, as $\frac{1}{k}I\left(S_{\epsilon }^{\left(k\right)},{\hat{S}}_{\epsilon }^{\left(k\right)}\right)=E\left\{Z_{k,\epsilon }^{\prime }\left(F_{S_{\epsilon },{\hat{S}}_{\epsilon }}^{\prime }\right)\right\}$([5] Equation (2.3)), we conclude that

(A38) $$R_{\epsilon }\left(D\right)\geq \underset{k\to \infty }{\lim\sup}E\left\{Z_{k,\epsilon }^{\prime }\left(F_{S_{\epsilon },{\hat{S}}_{\epsilon }}^{\prime }\right)\right\}.$$

Next, we consider $\underset{k\to \infty }{\lim\sup}E\left\{Z_{k_{l},\epsilon }^{\prime }\left(F_{S_{\epsilon },{\hat{S}}_{\epsilon }}^{\prime }\right)\right\}$: Let $Z_{k_{l},\epsilon }^{\prime }\left(F_{S_{\epsilon },{\hat{S}}_{\epsilon }}^{\prime }\right)$ be a subsequence of $E\left\{Z_{k,\epsilon }^{\prime }\left(F_{S_{\epsilon },{\hat{S}}_{\epsilon }}^{\prime }\right)\right\}$ with the indexes $k_{1}<k_{2}<\ldots$ such that its limit equals the limit superior. i.e., $\lim_{l\to \infty }E\left\{Z_{k_{l},\epsilon }^{\prime }\left(F_{S_{\epsilon },{\hat{S}}_{\epsilon }}^{\prime }\right)\right\}=\underset{k\to \infty }{\lim\sup}E\left\{Z_{k,\epsilon }^{\prime }\left(F_{S_{\epsilon },{\hat{S}}_{\epsilon }}^{\prime }\right)\right\}$. Since by Lemma A4, the sequence of non-negative RVs ${\left\{{\tilde{Z}}_{k_{l},n}^{\prime }\left(F_{S_{n},{\hat{S}}_{n}}^{\mathrm{opt}}\right)\right\}}_{n\in N}$ convergences in distribution to $Z_{k_{l},\epsilon }^{\prime }\left(F_{S_{\epsilon },{\hat{S}}_{\epsilon }}^{\prime }\right)$ as n→∞ uniformly in k∈N, it follows from ([40] Theorem 3.5) that $E\left\{Z_{k_{l},\epsilon }^{\prime }\left(F_{S_{\epsilon },{\hat{S}}_{\epsilon }}^{\prime }\right)\right\}=\lim_{n\to \infty }E\left\{{\tilde{Z}}_{k_{l},n}^{\prime }\left(F_{S_{n},{\hat{S}}_{n}}^{\mathrm{opt}}\right)\right\}$. Moreover, we define a family of distributions F(D) such that $F\left(D\right)=\left\{F_{S,\hat{S}}:D\left(F_{S,\hat{S}}\right)\leq D\right\}$. Consequently, Equation (A38) can now be written as:

(A39) $$\begin{array}{cc} R_{\epsilon }\left(D\right)\geq \underset{k\to \infty }{\lim\sup}E\left\{Z_{k,\epsilon }^{\prime }\left(F_{S_{\epsilon },{\hat{S}}_{\epsilon }}^{\prime }\right)\right\} & =\lim_{l\to \infty }\lim_{n\to \infty }E\left\{{\tilde{Z}}_{k_{l},n}^{\prime }\left(F_{S_{n},{\hat{S}}_{n}}^{\mathrm{opt}}\right)\right\}\\ \; & \overset{\left(a\right)}{=}\lim_{n\to \infty }\lim_{l\to \infty }E\left\{{\tilde{Z}}_{k_{l},n}^{\prime }\left(F_{S_{n},{\hat{S}}_{n}}^{\mathrm{opt}}\right)\right\}\\ \; & \overset{\left(b\right)}{=}\underset{n\to \infty }{\lim\sup}\lim_{l\to \infty }E\left\{{\tilde{Z}}_{k_{l},n}^{\prime }\left(F_{S_{n},{\hat{S}}_{n}}^{\mathrm{opt}}\right)\right\}\\ \; & \geq \underset{n\to \infty }{\lim\sup}\lim_{l\to \infty }\underset{F_{S,\hat{S}}\in F\left(D\right)}{\inf}E\left\{{\tilde{Z}}_{k_{l},n}^{\prime }\left(F_{S,\hat{S}}\right)\right\}\\ \; & \overset{\left(c\right)}{=}\underset{n\to \infty }{\lim\sup}\lim_{l\to \infty }\underset{F_{S,\hat{S}}\in F\left(D\right)}{\inf}\frac{1}{k_{l}}I\left({\hat{S}}_{n}^{\left(k_{l}\right)};S_{n}^{\left(k_{l}\right)}\right), \end{array}$$

where (a) follows since the convergence ${\tilde{Z}}_{k_{l},n}^{\prime }\left(F_{S_{n},{\hat{S}}_{n}}^{\mathrm{opt}}\right)\overset{\left(dist.\right)}{\underset{n\to \infty }{⟶}}Z_{k_{l},\epsilon }^{\prime }\left(F_{S_{\epsilon },\hat{S}}^{\prime }\right)$ is uniform with respect to $k_{l}$, thus the limits are interchangeable ([31] Theorem 7.11); (b) follows since the limit of the subsequence $E\left\{{\tilde{Z}}_{k_{l},n}^{\prime }\left(F_{S_{n},{\hat{S}}_{n}}^{\mathrm{opt}}\right)\right\}$ exists in the index *n*, and is therefore equivalent to the limit superior, $\underset{n\to \infty }{\lim\sup}E\left\{{\tilde{Z}}_{k_{l},n}^{\prime }\left(F_{S_{n},{\hat{S}}_{n}}^{\mathrm{opt}}\right)\right\}$ ([31] Page 57); and (c) holds since mutual information is the expected value of the mutual information density rate ([5] Equation (2.30)). Finally, we recall that in the proof of Lemma A5 it was established that the backward channel for the RDF at the distortion constraint *D*, defined in (21), is information stable, hence for such backward channels, we have from ([41] Theorem 1) that the minimum rate is defined as $R_{n}\left(D\right)=\lim_{k\to \infty }\underset{F_{S,\hat{S}}\in F\left(D\right)}{\inf}\frac{1}{k}I\left({\hat{S}}_{\epsilon }^{\left(k\right)};S_{n}^{\left(k\right)}\right)$ and the limit exists; Hence, $\lim_{k\to \infty }\underset{F_{S,\hat{S}}\in F\left(D\right)}{\inf}\frac{1}{k}I\left({\hat{S}}_{\epsilon }^{\left(k\right)};S_{n}^{\left(k\right)}\right)=\lim_{l\to \infty }\underset{F_{S,\hat{S}}\in F\left(D\right)}{\inf}\frac{1}{k_{l}}I\left({\hat{S}}^{\left(k_{l}\right)};S_{n}^{\left(k_{l}\right)}\right)$ in the index *k*. Substituting this into Equation (A39) yields the result:

(A40) $$R_{\epsilon }\left(D\right)\geq \underset{n\to \infty }{\lim\sup}R_{n}\left(D\right).$$

This proves the lemma. □

Combining the Lemmas A7 and A8 proves that $R_{\epsilon }\left(D\right)=\underset{n\to \infty }{\lim\sup}R_{n}\left(D\right)$ and the rate is achievable with Gaussian inputs, completing the proof of the theorem.
